# Characterization of the Regulatory AAA-ATPase Subunit Rpt3 in *Plasmodium berghei* as an Activator of Protein Phosphatase 1

**DOI:** 10.3390/ijms262311720

**Published:** 2025-12-03

**Authors:** Claudianne Lainé, Caroline De Witte, Alain Martoriati, Amaury Farce, Inès Metatla, Ida Chiara Guerrera, Katia Cailliau, Jamal Khalife, Christine Pierrot

**Affiliations:** 1 Univ. Lille, CNRS, Inserm, CHU Lille, Institut Pasteur de Lille, U1019-UMR 9017-CIIL-Center for Infection and Immunity of Lille, F-59000 Lille, Francecaroline.de-witte@pasteur-lille.fr (C.D.W.); jamal.khalife@pasteur-lille.fr (J.K.); 2 Univ. Lille, CNRS, UMR 8576-UGSF-Unité de Glycobiologie Structurale et Fonctionnelle, F-59000 Lille, France; alain.martoriati@univ-lille.fr (A.M.); katia.maggio@univ-lille.fr (K.C.); 3 Univ. Lille, Inserm, CHU Lille, U1286-Infinite-Institute for Translational Research in Inflammation, F-59000 Lille, France; amaury.farce@univ-lille.fr; 4 Proteomics Platform 3P5-Necker, Université Paris Descartes-Structure Fédérative de Recherche Necker, INSERM US24/CNRS UMS3633, 75015 Paris, France; ines.metatla@inserm.fr (I.M.); chiara.guerrera@inserm.fr (I.C.G.)

**Keywords:** proteasome, 19S regulatory particle, Rpt3, *Plasmodium*, Protein Phosphatase 1

## Abstract

The 26S proteasome is the main proteolytic machinery involved in protein degradation, thereby contributing to the homeostasis and stress response of eukaryotic cells. This macromolecular complex consists of a 20S core particle assembled with one or two 19S regulatory particles. Here, we describe the *Plasmodium berghei* (Pb) proteasome AAA-ATPase regulatory subunit Rpt3 and demonstrate its binding to the Protein Phosphatase 1 catalytic subunit (PP1c), which is one of the major and essential parasite phosphatases. The PbRpt3 protein enhances the activity of PP1c both in vitro and in a *Xenopus* oocyte heterologous model. Further investigation of this model suggests that the PbRpt3-PP1c interaction may occur outside of the proteasome, and it reveals that the RVxF motifs of PbRpt3 are involved in its binding and regulatory function. Moreover, the ATP-binding capacity of PbRpt3 may also contribute to its phosphatase regulatory activity. In the parasite, reverse genetic studies suggest an essential role for PbRpt3 during erythrocytic cycle of *P. berghei*, and an interactome analysis confirmed that PbRpt3 belongs to the 19S regulatory particle of the proteasome and may interact with proteins previously shown to be involved in phospholipid binding.

## 1. Introduction

The ubiquitin-proteasome system is one of the main pathways in protein degradation. Its cellular functions range from general cellular homeostasis and stress response to the control of vital processes such as cell division and signal transduction. The proteasome is a large multi-protein enzyme complex found in eukaryotes, archaea, and some bacteria of the order Actinomycetales (for review [1]). In eukaryotic cells, it is constitutively present in the cytosol, associated with the endoplasmic reticulum, and can also be present in the nucleus, depending on the cell type, growth status, and whether the cell is encountering stimulation or stress [2,3,4]. As the endpoint for the ubiquitin-proteasome system, the 26S proteasome is the principal proteolytic machinery responsible for protein degradation of misfolded, denatured, or obsolete proteins by proteolysis.

In *Plasmodium*, the proteasome 26S has been purified and characterized [5,6]. This parasite, causative agent of malaria, undergoes rapid growth and cell division in an oxidatively stressed environment. Consequently, it is highly dependent on tight and quick protein turnover machinery. Interestingly, proteasome inhibitors exhibit parasiticidal activity at different stages of the parasite’s lifecycle (for review [7]). Moreover, inhibitors of the proteasome strongly synergize artemisinin-induced killing of *Plasmodium*, both in vitro and in vivo [8,9], underscoring the essentiality of the proteasome function in this parasite.

Similar to eukaryotes, *Plasmodium* 26S proteasome comprises a 19S regulatory particle and a 20S core particle. The structure of the 20S has been characterized using high-resolution electron cryo-microscopy, thereby establishing the molecular basis of the parasite 20S proteasome specificity when compared to its human counterpart [10]. The 19S is composed of two subcomplexes: the base, which directly binds to the 20S, and the lid [11]. The base is formed by six AAA-ATPase subunits (Rpt1-6), and four non-ATPase subunits (Rpn1, Rpn2, Rpn10, and Rpn13). The other nine non-ATPase subunits constitute the lid. The role of the 19S is to recognize and unfold the polyubiquitinated substrates and open the channel prior to the substrate translocation into the 20S cavity. All these activities except for substrate recognition depend on ATP binding/hydrolysis by the ATPase subunits. The core 20S particle forms a cylinder that houses the proteolytic activity of the proteasome within a central chamber [12]. It is composed of two related types of subunits: α subunits, which form the outer two heptameric rings, and β subunits, which form the inner pair of heptameric rings and include the proteolytic active sites.

Many proteasome-interacting proteins have been identified and regulate its assembly and activity. In *Plasmodium falciparum* (Pf), a study aimed at isolating the functional 26S proteasome revealed that the proteasome co-purified proteins involved in biosynthesis, folding, quality control, and degradation [5]. Among these, chaperone proteins such as heat shock proteins and the TCP-1 complex were identified, suggesting their possible roles in regulating protein degradation through interactions with the proteasome. More recently, our analyses based on a proteomic approach conducted in *Plasmodium berghei* (Pb) have enabled us to identify some of the 19S AAA-ATPase and non-ATPase subunits as potential interactors of the parasite Protein Phosphatase 1 catalytic subunit (PP1c) [13,14]. PP1 is a key enzyme in *Plasmodium*. This phosphatase seems to be responsible for the majority of the dephosphorylation processes in the parasite [15], and reverse genetic analyses have demonstrated its essential role in blood-stage parasite development and egress from erythrocytes [16,17,18]. PP1 is a multifunctional holoenzyme consisting of a highly conserved catalytic subunit (PP1c) along with one or more regulatory subunits which direct its localization and shape its activity/specificity [19,20,21,22,23]. A common feature of most of the PP1c regulators is that they contain a RVxF sequence as the primary PP1c-binding motif [24]. In *Plasmodium*, numerous potential partners have been identified in global analyses of PP1c interactome [13,14]. This includes both conserved and specific regulators [13,25,26,27,28]. The identification of some components of the 19S proteasome among the PbPP1c-binding proteins suggests that they could be PP1c substrates, recruit additional substrates, modify the localization of the phosphatase, and/or regulate its activity.

To gain a deeper insight into the relationship between the proteasome and PP1c, we conducted a molecular and functional characterization of PbRpt3, an AAA-ATPase subunit of the *P. berghei* 19S that we identified as a potential interactor of PbPP1c [13,14]. Here, we show that PbRpt3 binds to PP1c and increases the phosphatase activity both in vitro and in the heterologous model of *Xenopus* oocytes. Using site-directed mutagenesis, we demonstrated that the PbRpt3 RVxF motifs play a role in this interaction and function, and we provided evidence that the binding of ATP is involved in this regulatory activity. Our PbRpt3 model, combined with experiments in *Xenopus* oocytes, predicted an interaction between PbRpt3 and PP1c outside the proteasome. To assess the effect of Rpt3 on PP1c activity within the parasite, we aimed to generate PbRpt3 knockout parasites. However, all our attempts to obtain viable, cloned parasites were unsuccessful, indicating that PbRpt3 plays a critical role in either proteasome assembly or PP1c function. Finally, immunoprecipitation of tagged PbRpt3 followed by mass spectrometry analysis confirmed that the PbRpt3-PP1c interaction may occur outside the proteasome complex and identified many potential PbRpt3 interactors, including proteins involved in membrane dynamics.

## 2. Results and Discussion

### 2.1. PbRpt3 Sequence Analysis and Protein Annotation

**Figure 1:**
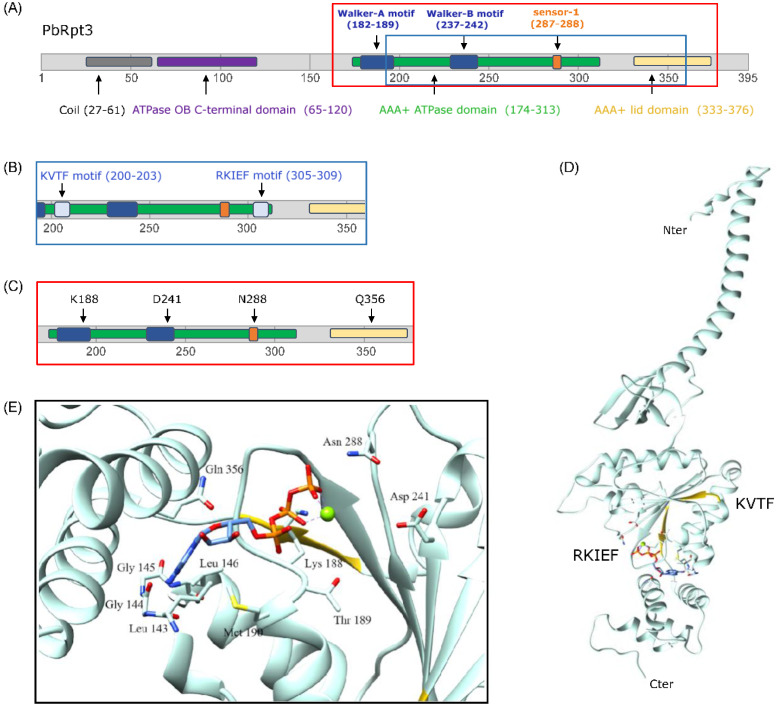
*P. berghei* regulatory AAA-ATPase subunit PbRpt3 domains and structural model. (**A**) Prediction of the PbRpt3 domains using InterproScan software (Version 5.61) combined with visual inspection. Schematic of the PbRpt3 domains showing (**B**) part of the AAA-ATPase domain corresponding to the two RVxF consensus sequences: KVTF and RKIEF and (**C**) part of the AAA-ATPase domain where four amino acids are located: K188, D241, N288, and Q356, which are essential for Mg^2+^ and ATP binding. (**D**) Predicted tertiary structure of PbRpt3. Image generated from PDB file 6MSB P auth D chain predicted with Sybyl (Version 6.9.2, Tripos) and annotated with Chimera (Version 35), highlighting the two RVXF motifs (in yellow) and the ATP molecule at the center. (**E**) Inset shows the side structure of the amino acid chains involved in ATP-binding and Mg^2+^ stabilization: the ATP molecule is modelled next to the Mg^2+^ ion (green) and surrounded by K188 and Q356, which are the main holders of the molecule.

Exploration of PlasmoDB database showed that PbRpt3 gene (PBANKA_0715600) is predicted to span 1516 bp, including an intron of 328 bp. The sequencing of the PCR products obtained with specific primers on both cDNA and genomic DNA confirmed both the transcription of PbRpt3 and its correct exons-introns positions, respectively (data not shown). The deduced sequence of 395 amino acids (a.a.) (Supplementary Figure S1) showed 100% identity to the PlasmoDB predicted sequence. Although the *Plasmodium* protein is 23 a.a. shorter than its human counterpart PSMC4 (Supplementary Figure S2), it exhibits conserved motifs and domains. Indeed, InterproScan analysis of the potential domains of PbRpt3, along with visual inspection, identified an AAA-ATPase domain with a Walker A motif (consensus G-x(4)-GK-[TS] and sequence GPPGTGKT) at position 182 as well as a Walker B motif (consensus hhhDE and sequence IIFIDE) at position 237 (Figure 1A and Figure S1). These motifs, also present in human Rpt, are common features of AAA+ proteins and are critical for nucleotide binding and hydrolysis by coordinating the β- and γ-phosphates of ATP and a magnesium ion [29,30]. The PbRpt3 a.a. sequence also shows a sensor-1 motif (TN at position 287) including a conserved arginine, which is described to hydrogen bond with the γ phosphate of ATP [31]. A coil domain is predicted at the Nter of PbRpt3. Coil domains have been previously described to be involved in the coiled-coil interaction between the different Rpt proteins of the proteasome 19S complex [32]. Furthermore, a.a. sequence analysis showed the presence of two consensus RVxF motifs, well known as the PP1c binding motif. These two motifs, at positions 200 (200-KVTF-203) and 305 (305-RKIEF-309), fit to the consensus sequence [R/K]-X(0-1)-[V/I]-{P}-[F/W] ({P} being any amino acid except a proline) (Figure 1B). The first motif KVTF is not conserved between all *Plasmodium* species (Supplementary Table S1). It is present in *Plasmodium knowlesi* Rpt3 but absent in *P. falciparum* Rpt3 (PF3D7_0413600) and three other *Plasmodium* species infecting humans. Regarding the second motif RKIEF, it is conserved among all *Plasmodium* species as well as among mammals (*Homo sapiens*, *Mus musculus*), yeast (*Saccharomyces cerevisiae*), and plants (*Arabidopsis thaliana—*Uniprot Reference A0A178UAH2). The presence of two putative RVxF motifs in PbRpt3 may explain its detection in PbPP1c interactome [13,14] and likely suggests that it could be a direct interactor of the phosphatase.

### 2.2. PbRpt3 Exhibits ATP-Binding Ability and Would Require a Mg^2+^ Ion for Complex Stability

In order to examine the sequence/structure relationship of PbRpt3, a 3D model was predicted (Figure 1D) based on the cryomicroscopic structure of the human Rpt3 (PSMC4 isoform 1 of 418 residues), as these two proteins share a sequence identity of 67% (Supplementary Figure S2). As expected, the 3D structure showed a coil region in its N-terminus, an OB-C-terminal domain (a.a. 64 to 119) including a five-stranded β-sheet, with a very short α helix positioned between the third and fourth strands. This is followed by the ATPase domain comprising an α-β-α subdomain, which includes the motifs involved in ATP-binding and hydrolysis (the Walker A motif is located between a β-sheet and an α helix, and the Walker B motif forms a whole β-sheet). The AAA-lid domain is also present and reveals an all-α helix subdomain with four α helix between a.a. 320–360. Interestingly, our 3D model predicted that an Mg^2+^ ion would be required to stabilize the PbRpt3 structure, as reported for the human Rpt3 (PSMC4) [33]. In silico docking simulation of the ATP on our 3D model showed that this molecule would mainly be maintained with hydrogen bonds with the K188 and the Q356 of the protein (Figure 1E). N288 would also contribute to stabilizing ATP through hydrogen bonds. In addition, ATP appears to be essential for the maintenance of the magnesium ion, and D241 would contribute to the stabilization of this ion. Thus, the four amino acids K188, D241, N288, and Q356 seem to play an important role in stabilizing the ATP molecule and, in extenso, they are important for the binding of Mg^2+^ and the structural arrangement of the protein. Of note, K188 and D241 are located in the Walker A and Walker B motifs, respectively (Figure 1C). Both K188 and D241 correspond to conserved residues whose mutations have been shown to abolish ATP-binding and hydrolysis, respectively [34,35]. In addition, the asparagine at position 288 corresponds to the conserved hydrogen-bonding polar residue of the sensor-1 motif, which is known to position a water molecule relative to the γ phosphate of ATP, thereby facilitating nucleotide hydrolysis [36]. The identification of these three residues in our ATP docking simulation at conserved positions in the protein structures (Supplementary Figures S3 and S4) further validates our homology model. Another part of the protein backbone of PbRpt3 would also contribute to the stabilization of ATP with the sequence 143-LGG-145. However, the mutation of these amino acids to alanine residues would not change the conformational structure of the pocket where the adenine group of ATP is located (data not shown).

### 2.3. PbRpt3 Could Interact with PP1c Independently of the Proteasome Complex

The 3D predicted model of PbRpt3 showed that the two RVxF motifs (200-KVTF-203 and 305-RKIEF-309) are located on either side of the protein, within the AAA-ATPase domain (Figure 1B,D and Figure S5A). This accessible location would allow PbRpt3 to interact with PP1c, an interaction that could involve either of the two RVxF motifs. Indeed, the folding of PfPP1c has been modeled (https://swissmodel.expasy.org/repository/uniprot/Q8ILV1?csm=9CE0B7C29FEFEFC6 (accessed on 12 April 2023)), and it appears that its compact predicted conformation [37] would not allow the interaction with both PbRpt3 RVxF motifs simultaneously.

To better explore how the 3D model of PbRpt3 protein fits in the proteasome complex, and to assess the accessibility of the RVxF motifs of PbRpt3 for a possible interaction with PbPP1c, we replaced the experimental human PSMC4 by the modeled 3D structure of PbRpt3 within the crystallographic structure of the human proteasome (RCSB PDB, ID 6MSB) (Supplementary Figure S5B). Analysis of this integration using the Chimera software (Version 35) revealed that the immediate environment of the RVxF motifs of PbRpt3 includes the B chain PSMC1 (Rpt2), the F chain PSMC3 (Rpt5), the K and L chains (alpha subunits), and the f chain PSMD2 (Rpn1). This renders the motif inaccessible for PP1c interaction. Regarding the KVTF motif, only Lys 200 would be accessible. However, phenylalanine residue of RVxF motif is known to be crucial for the interaction with PP1c [19]. Consequently, if all the proteasome protein–protein interactions are conserved in *P. berghei* as in humans, the interaction between PbRpt3 and PbPP1c could potentially occur when PbRpt3 is not tethered to the 26S proteasome complex, possibly before or after its assembly and/or disassembly.

### 2.4. His-Tagged PbRpt3 Protein Directly Interacts with PP1c and Regulates Its Activity

**Figure 2:**
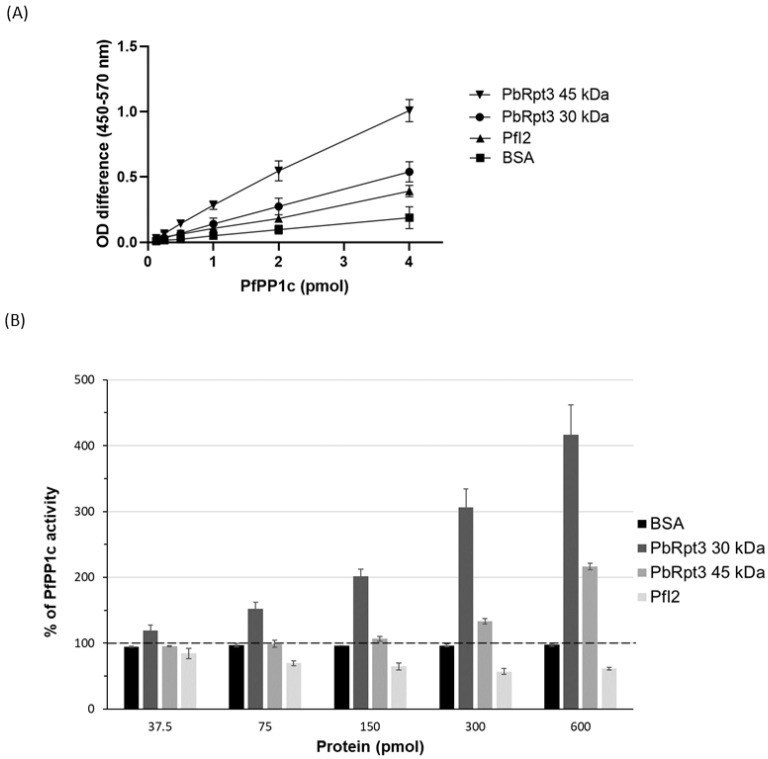
Interaction of PbRpt3 with PP1c in vitro and effect on phosphatase activity. (**A**) Interaction study of PbRpt3−45 kDa and PbRpt3−30 kDa with PfPP1c using PfI2 as a positive control and BSA as a negative control. Results are representative of two independent experiments. (**B**) Effect of PbRpt3−45 kDa, PbRpt3−30 kDa, BSA, and PfI2 on PfPP1c phosphatase activity. Different quantities of each protein (37.5, 75, 150, 300, and 600 pmol) were incubated with 1 µg of recombinant PfPP1c for 30 min before the incubation with the substrate pNPP for 1 h at 37 °C. Results presented as % of relative increase or decrease of phosphatase activity are means ± S.D. of two independent experiments performed in duplicate.

The interactome of PbPP1c showed that PbRpt3 was identified in immunoprecipitated protein complexes based on mass spectrometry analysis [13,14]. In order to investigate whether these proteins interact directly, we produced a recombinant protein corresponding to the PbRpt3 full length (aa 1–395, 45 kDa, Supplementary Figure S6A) and performed an ELISA-based assay using biotinylated PP1c. In these experiments, PfI2 a well-known direct partner and inhibitor of PP1c, was used as a control [27]. The results shown in Figure 2A demonstrate that PbRpt3-45 kDa directly interacts with PfPP1c in a dose-dependent manner. To determine further which region of PbRpt3 interacts with PP1c, we produced a recombinant truncated PbRpt3 (aa 131–395, 30 kDa, Supplementary Figure S6B). This fragment lacks the coil region and the ATPase OB-C-terminal domain. Interestingly, this truncated PbRpt3 also interacts with PfPP1c in a concentration-dependent manner in ELISA-based assay (Figure 2A). Taken together, these results suggest that the PP1c-interaction domains of PbRpt3 may lie in this region where both RVxF motifs are present.

We then assessed the effect of PbRpt3 on PP1c phosphatase activity using p-nitrophenyl phosphate (pNPP) as the substrate. First, a control experiment showed that when pNPP was used as substrate in the presence of PbRpt3 alone (without PfPP1c), no phosphatase activity was detected (data not shown). In the presence of PfPP1c, the recombinant protein PbRpt3-45 kDa strongly increased the dephosphorylation activity of the phosphatase in a concentration-dependent manner (Figure 2B), with a mean value of 220% of PP1c activity when a quantity of 600 pmol of PbRpt3 was used. Similarly, the PbRpt3-30 kDa recombinant protein also increased PfPP1c activity, reaching more than 400% PP1c activity when incubated with 600 pmol of the truncated PbRpt3 (Figure 2B). As expected, PfI2 decreased PfPP1c activity (~60% of PfPP1c activity using 600 pmol of PfI2) [27]. These results strongly suggest that PbRpt3 would be an activator of PP1c. Moreover, although we cannot compare the values obtained with PbRpt3-45 kDa and PbRpt3-30 kDa due to a potential difference in the purity of the two recombinant proteins (Supplementary Figure S6), these results clearly indicate that the activity of PbRpt3 on PfPP1c is carried by the aa 131–395 region of the protein.

### 2.5. PbRpt3 Binds to PP1c Outside the Proteasome and Shows Functional Activity in Xenopus Oocytes

**Figure 3:**
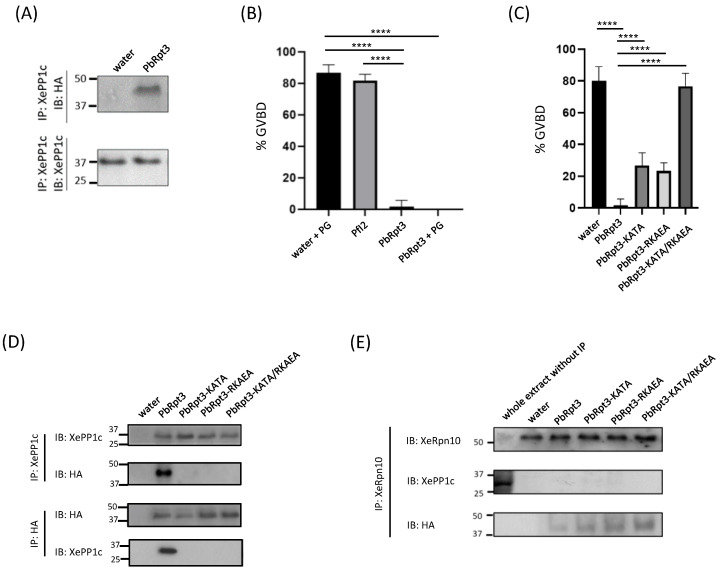
PbRpt3 functional analysis using *Xenopus* oocytes model. (**A**) Interaction of PbRpt3 with *Xenopus* PP1c (XePP1c). Extracts prepared from *Xenopus* oocytes previously micro-injected with either water (80 nL, lane 1) or PbRpt3 cRNA (60 ng in 80 nL, lane 2), followed by progesterone (PG) treatment (10 μM, 15 h after micro-injection) were immunoprecipitated using an anti-XePP1c mAb. An immunoblot analysis was performed using anti-HA mAb (upper panel) and anti-XePP1c mAb (lower panel). (**B**,**C**) Role of PbRpt3 on the induction of Germinal Vesicle BreakDown (GVBD). Values are presented as mean percentages. ****, *p* value < 0.0001. Each experiment was performed using a set of 10 oocytes and repeated on three animals. (**B**) The appearance of GVBD induced by PG 15 h after microinjection of water (60 nL as control, lane 1) is strongly inhibited by the micro-injection of PbRpt3 cRNA (20 ng in 60 nL, lane 4). In the absence of PG, the micro-injection of PbRpt3 cRNA (20 ng in 60 nL, lane 3) is not sufficient to trigger the GVBD. As a control, the microinjection of the PP1c inhibitor PfI2 cRNA (20 ng in 60 nL, lane 2) triggers the oocytes GVBD. (**C**) Role of the RVxF motifs of PbRpt3 for its functional activity. Percentage of GVBD of oocytes which have been micro-injected with either water as control (80 nL, lane 1), or cRNAs (60 ng in 80 nL) coding for PbRpt3 (lane 2), PbRpt3-KATA (lane 3), PbRpt3-RKAEA (lane 4), or PbRpt3-KATA/RKAEA (lane 5). All micro-injections were followed by PG treatment (10 μM, 15 h after micro-injection). (**D**) Role of the RVxF motifs of PbRpt3 for its interaction with XePP1c. Oocytes were micro-injected with water as control (80 nL, lane 1) or cRNAs (60 ng in 80 nL) coding for PbRpt3 (lane 2), PbRpt3-KATA (lane 3), PbRpt3-RKAEA (lane 4), or PbRpt3-KATA/RKAEA (lane 5), 15 h prior incubation with PG (10 μM). Then, the extracts were immunoprecipitated with anti-XePP1c mAb (upper panel) or anti-HA mAb (lower panel) and subjected to immunodetection using the same antibodies. Each experiment was performed using a set of 20 oocytes and repeated on two animals. (**E**) Interaction of PbRpt3 with *Xenopus* 19S proteasome subunit Rpn10. Oocytes were micro-injected with water (80 nL, lane 2) or cRNAs (60 ng in 80 nL) coding for PbRpt3 (lane 3), PbRpt3-KATA (lane 4), PbRpt3-RKAEA (lane 5), or PbRpt3-KATA/RKAEA (lane 6), 15 h prior incubation with PG (10 μM). Immunoprecipitation of oocytes extracts was performed with anti-XeRpn10 mAb, and complexes were immunodetected using the same antibodies (upper panel), anti-XePP1c mAb (middle panel) or anti-HA mAb (lower panel). As a control, whole oocytes extract without IP was included (lane 1). Each experiment was performed using a set of 20 oocytes and repeated on two animals.

To gain deeper insight into the functional activity of PbRpt3 in a cellular context, we took advantage of the *Xenopus* oocyte model in which the Rpt3 counterpart exhibits 66% identity with PbRpt3. This choice is also based on the fact that *Xenopus laevis* PP1c (XePP1c) shows >80% identity with PbPP1c [28]. Furthermore, in this model, it has been previously shown that several partners of PP1c could regulate cell-cycle progression from G2 to M, assessed by the appearance of GVBD (Germinal Vesicle Break Down) [25,27,28,38]. In the *Xenopus* model, immature oocytes are blocked in prophase I, and the inhibition of PP1c by anti-PP1c antibodies or by an inhibitor of this phosphatase will trigger the GVBD reflecting the G2/M transition to metaphase II [27,28,38,39]. Conversely, a PP1c activator will lead to an inhibition of the progesterone (PG)-induced maturation. To confirm the in vitro results showing the activation of PP1c by the recombinant PbRpt3, we microinjected the cRNA coding for the HA-tagged PbRpt3 protein (Supplementary Figure S7). First, the efficient translation of PbRpt3 in oocytes after cRNA microinjection was checked via immunoblot using anti-HA mAb (Supplementary Figure S8). Next, we performed immunoprecipitations of oocyte extracts using anti-*Xenopus* PP1c (XePP1c) mAb. The immunoblot analysis revealed that PbRpt3 and XePP1c were present in the same complex (Figure 3A). At the functional level, the microinjection of PbRpt3 cRNA alone did not induce the maturation of *Xenopus* oocyte, while PG or PfI2 did induce GVBD (Figure 3B). When oocytes maturation was induced with PG, we observed that the microinjection of PbRpt3 cRNA resulted in a significant reduction of the percentage of GVBD (Figure 3B; mean percentage of GVBD: 86.7% and 1.7% for PG and PG + PbRpt3 cRNA, respectively). Taken together, these results show that PbRpt3 seems to activate XePP1c in oocytes, confirming the results obtained in vitro.

To test whether this could occur when Rpt3 is assembled into the proteasomal complex, immunoprecipitation experiments were performed using an anti-*Xenopus* Rpn10 (XeRpn10) antibody. This allowed us to pull down PbRpt3-HA, but not XePP1c (Figure 3E, lane 3). Rpn10 is a subunit of the proteasome 19S regulatory particle and is described to act as a ubiquitin receptor, interacting with both base and lid subparticles [40,41,42]. Immunoprecipitation of PbRpt3 with XeRpn10 shows that the parasite protein can be incorporated into the 19S complex of the *Xenopus* oocyte. This is in alignment with the observed conservation of Rpt3 across species. Additionally, our results suggest that XePP1c is not part of the PbRpt3-Rpn10 complex, indicating that the Rpt3-PP1c interaction may occur outside the proteasome.

### 2.6. PbRpt3 RVxF Motifs Are Crucial for PP1c Binding and Functional Activity in Xenopus Oocytes

Many studies showed that the binding of PP1c to RVxF-dependent interacting proteins could be disrupted when their RVxF amino acids V/I or F are mutated to alanine residues [43,44]. In order to explore the contribution of RVxF motifs of PbRpt3 in its functional activity, we generated PbRpt3 cRNAs bearing single-motif mutations either on 200-KVTF-203 (PbRpt3-KATA) or 305-RKIEF-309 (PbRpt3-RKAEA), or double-motif mutations (PbRpt3-KATA/RKAEA) (Supplementary Figure S7). The effective translation in the oocytes of each microinjected cRNA was checked via immunoblot using anti-HA mAb (Supplementary Figure S8). We then assessed the capacity of each single mutated protein as well as the double-mutated protein to inhibit the PG-induced GVBD. As with PbRpt3, the microinjection of either single mutated cRNA, PbRpt3-KATA, or PbRpt3-RKAEA resulted in a significant reduction in the PG-induced GVBD (Figure 3C, lanes 3 and 4). However, this inhibition seemed to be partial when single mutated cRNAs were microinjected (mean percentage of GVBD of 80%, 1.7%, 26.6%, and 23.3% for control, PbRpt3, PbRpt3-KATA, or PbRpt3-RKAEA, respectively). The co-immunoprecipitation/immunoblot assays performed on lysates prepared from these oocytes indicated that the PbRpt3-XePP1c complex was not detected when single mutants were injected (Figure 3D). The partial functional effect of single mutants may thus be attributed to a residual and/or transitory binding of PbRpt3 to XePP1c that would not be detected by immunoblot. To further define the contribution of both RVxF motifs, we microinjected the double-mutated cRNA (PbRpt3-KATA/RKAEA) in the oocytes and followed up the appearance of GVBD. As shown in Figure 3C, this double mutant could not prevent PG-induced GVBD (lane 5; percentage of GVBD 76%). In addition, PbRpt3-KATA/RKAEA mutant protein failed to co-immunoprecipitate with XePP1c (Figure 3D, lane 5), despite its effective translation by the oocyte (Supplementary Figure S8, lane 6). Taken together, these results demonstrate that PbRpt3 directly interacts with PP1c, and that its interaction with PP1c via both RVxF motifs would be required for its functional effect.

### 2.7. The ATP-Binding Capacity of PbRpt3 Is Involved in the Activation of XePP1c

**Figure 4:**
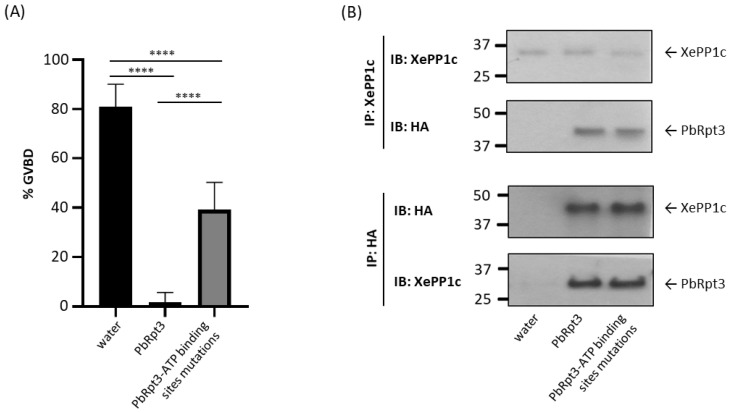
Role of the predicted ATP-binding sites of PbRpt3 in its functional activity in *Xenopus* oocytes. (**A**) Percentage of GVBD of oocytes which have been micro-injected with either water as control (80 nL, lane 1), PbRpt3 cRNA (60 ng in 80 nL, lane 2), and PbRpt3 cRNA mutated on 4 ATP-binding sites (60 ng in 80 nL, lane 3). All micro-injections were followed by progesterone treatment (10 μM, 15 h after micro-injection). This experiment was performed using 40 oocytes from two independent animals and reproduced on a total of 100 oocytes micro-injected with three different batches of cRNA. Values are presented as mean percentages. ****, *p* value < 0.0001. (**B**) Interaction between XePP1c and PbRpt3 analyzed by immunoprecipitation. Twenty oocytes were micro-injected with water as control (80 nL, lane 1), PbRpt3 cRNA (60 ng in 80 nL, lane 2) and PbRpt3 cRNA mutated on four ATP-binding sites (60 ng in 80 nL, lane 3), 15 h prior incubation with PG (10 μM). The extracts were immunoprecipitated with anti-XePP1c mAb (upper panel) or anti-HA mAb (lower panel) and subjected to immunodetection using the same mAbs. Each experiment was repeated on two animals.

PbRpt3 is expected to be an AAA-ATPase, as evidenced by the domain present in its protein sequence. In the predicted 3D structure, we observed that four amino acids (K188, D241, N288, and Q356) are involved in stabilizing the ATP molecule, as well as in the binding of a Mg^2+^ ion. The mutation of these amino acids may thus disrupt the enzymatic activity of the protein. To explore whether the binding of ATP to PbRpt3 is involved in its functional activity towards PP1c in *Xenopus* oocytes, we generated a mutated cRNA for PbRpt3 where all the four residues, K188, D241, N288, and Q356 (Supplementary Figure S7) were replaced by an alanine. The results in Figure 4 showed that the mutation of these amino acids partially abolished the inhibition of PG-induced GVBD by PbRpt3. The microinjection of the PbRpt3 cRNA mutated for the ATP-binding sites resulted in a 39.2% GVBD, while PbRpt3 cRNA microinjection resulted in 1.7% GVBD (mean percentage of GVBD in PG-treated oocytes: 80.8%). Interestingly, we observed that in the subsequent immunoprecipitation experiments, the mutated PbRpt3 protein conserved its binding capacity to XePP1c (Figure 4B, lane 3). The partial effect observed on PG-induced GVBD could be explained either by a partial contribution of the ATP-binding capacity of PbRpt3, or by a possible residual capacity of the mutated protein to bind an ATP molecule. In fact, as described above, the sequence 143-LGG-145 may be involved in stabilizing the ATP even in the absence of certain crucial residues granting the presence of stabilizing hydrogen bounds. However, structural modelling predicted that replacing these three amino acids with alanine residues would not directly affect the interaction of the lateral chains with ATP or the organization of the protein backbone in this region, thus indicating that the ATP-maintaining pocket would be highly stable. Moreover, in vitro assays using pNPP as a substrate did not reveal any effect of varying concentrations of ADP or ATP on the capacity of recombinant PbRpt3 to activate PP1c (data not shown). While this suggests that binding of ATP may not be involved in PbRpt3’s role in activating pNPP dephosphorylation by PP1c in vitro, our findings in *Xenopus* oocytes indicate for the first time that the ATP-binding of PbRpt3 may play a role in activating the phosphatase PP1c in a cellular context.

### 2.8. PbRpt3 May Be Essential During the Blood Stage Life Cycle of P. berghei

The data presented above indicate that PbRpt3 interacts with PP1c and enhances its activity. Next, we aimed to assess the effect of Rpt3 on PP1c activity in *P. berghei* by examining the consequences of its deletion on phosphoproteomic profile. This investigation was also motivated by a previous high-throughput functional analysis that suggested deleting PbRpt3 results in viable parasites with a “slow-growth” phenotype compared to the parental strain [45]. Therefore, we attempted to generate knock-out (KO) lines by taking advantage of the available PlasmoGEM plasmid (PbGEM-022521; 93% deletion of the PbRpt3 gene). Transfections were performed in two different strains of *P. berghei* ANKA (pG230 and PbGFP) with this KOgem construct. After selection with pyrimethamine, resistant parasites were genotyped via PCR (see Supplementary Table S2 and Supplementary Figure S9A). A total of four independent transfections were performed, allowing the detection of parasites seven to nine days after transfection and pyrimethamine selection. In two of these transfections, we detected the integration of the resistance cassette on 5’ and 3′ sides, as well as the endogenous PbRpt3 gene (Supplementary Figure S9B). We then tried cloning these parasites by limiting dilution. After five attempts with different amounts of parasites injected per mouse, we only obtained either a mixture of transgenic and wild-type parasites or pure wild-type parasites (Supplementary Figure S9C). These findings support the crucial role of PbRpt3 during the erythrocytic cycle of *P. berghei* and seem to contradict the previous study in which a “slow-growth” rate of PbRpt3-KO parasites was observed [45]. However, it is important to consider that in this large-scale functional screening, mutants were obtained through simultaneous co-transfection of multiple barcode vectors, and parasite growth was followed in uncloned population, which could potentially account for this discrepancy. In *P. falciparum*, a saturation mutagenesis study showed that most of the proteasome 26S genes, including PfRpt3, were essential for parasite survival [46]. In addition, selective inhibitors of the catalytic subunits of the parasite proteasome have shown potent anti-malarial effects [7,8,47]. Thus, the essentiality of PbRpt3 could be related either to its role in the proteasome complex and/or to its ability to bind and regulate the essential PP1c enzyme in *Plasmodium* [16,17].

### 2.9. Localization of PbRpt3 in P. berghei

**Figure 5:**
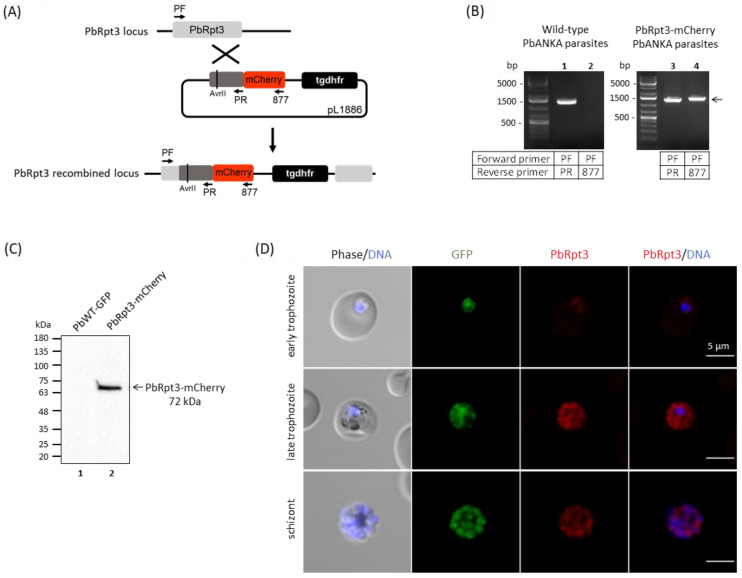
Tagging of endogenous Rpt3 in *P. berghei* and localization studies (**A**) Knock-in strategy using the vector pL1886 that allows the insertion of the mCherry epitope (in red) at the C-terminus of PbRpt3 with a single homologous recombination. The pL1886 construct, the tgdhfr-resistance cassette, the location of the primers used for PCR analysis, and the locus resulting from integration are shown. A silent mutation was inserted in the coding sequence to generate an AvrII single cutter restriction site in order to linearize the plasmid before transfection into PbANKA-GFP strain. (**B**) Diagnosis PCR analysis of pL1886-tgdhfr-PbRpt3-mCherry transfected *P. berghei* parasites. Lanes 1 and 2 correspond to DNA extracted from wild-type (WT) *P. berghei* ANKA parasite, and lanes 3 and 4 correspond to DNA extracted from transfected parasites. Lanes 1 and 3 represent the detection of the wild-type locus (PCR with PF and PR), and lanes 2 and 4 correspond to the detection of the 5′ integration at the PbRpt3 locus (PCR with PF-877, arrow). (**C**) Immunoblot analysis of pL1886-PbRpt3-mCherry transfected *P. berghei*. Proteins extracted from wild type parasites (lane 1) or from transfected parasites (lane 2) were subjected to Western blotting, probed with anti-RFP antibodies. (**D**). Cellular distribution of PbRpt3-mCherry in *P. berghei* erythrocytic stages (early trophozoite, late trophozoite and schizont), analyzed via immunofluorescence with anti-mCherry antibodies. The PbRpt3 protein appears in red, and the DNA is stained with DAPI. The parasites express the Green Fluorescent Protein (GFP). Scale bar = 5 μm.

In order to identify the interaction networks of PbRpt3 in *P. berghei,* we generated parasites expressing mCherry-tagged PbRpt3 using a single homologous strategy (Figure 5A). The correct integration of the mCherry tag was checked by PCR genotyping (Figure 5B). Following enrichment of the mCherry positive parasites by cell sorting, the expression of the tagged protein was checked via immunoblot analysis (Figure 5C). We took advantage of this parasite line to assess PbRpt3 localization using immunofluorescence assays. PbRpt3-mCherry expression was observed throughout the erythrocytic cycle of *P. berghei*, with a peak in late trophozoite stages (Figure 5D). This expression profile is consistent with the results of transcriptomic studies showing a maximum transcript level at the trophozoite and schizont stages [48]. Concerning the cellular distribution of PbRpt3-mCherry, we observed that it is mainly cytoplasmic in trophozoites and schizonts stages, with a punctuated localization particularly observed in late trophozoites (Figure 5D). This localization may correspond to parasites organelle structures or to proteasome storage granules. Interestingly, the latter have been described in the cytoplasm of quiescent yeasts [49]. Overall, the distribution of PbRpt3 fits with the localization of the proteasomes in eukaryotic cells as they are mainly found in the cytoplasm of the cell, associated with the centrosomes, the cytoskeletal networks, and the outer surface of the endoplasmic reticulum [3,4].

### 2.10. PbRpt3 Interacts with the 19S Proteasome Complex

**Figure 6:**
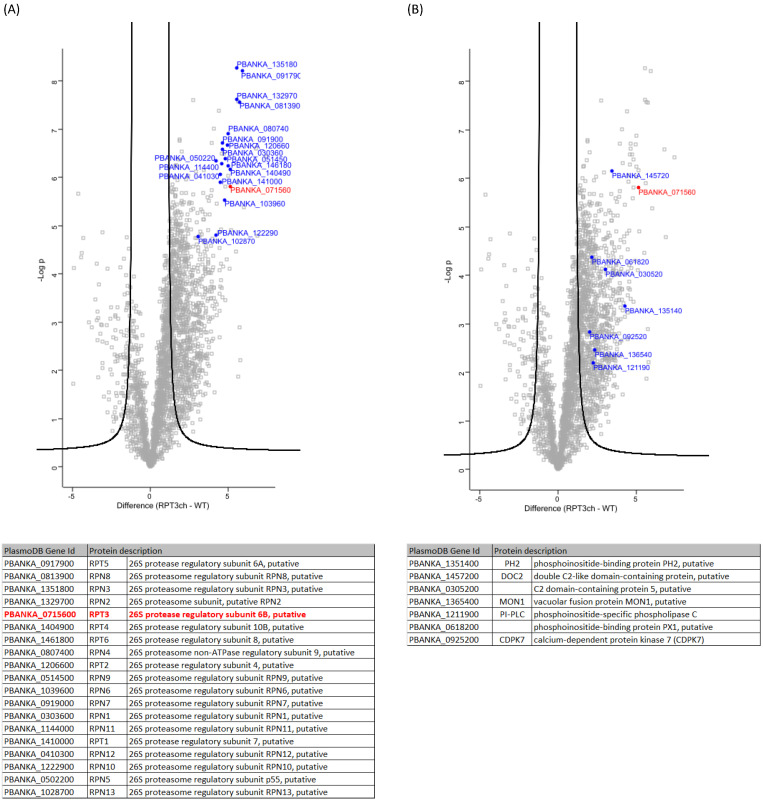
PbRpt3 interactome analysis. Volcano plot representations of the outcome of the PbRpt3 interactome study. PbRpt3 (PBANKA_0715600) is highlighted in red. Highlighted in blue are the interacting proteins belonging to the 19S proteasome (**A**) and to the GO enriched “phospholipid binding pathway” (**B**) List of the proteins is included in the tables below the plots, ranked according to their Student’s t-test difference. Gene identifiers (Id) and protein descriptions were updated using PlasmoDB relapse 68 (May 2024), except for Rpn4 and Rpt2, which have short names attributed based on sequence homology.

In order to identify the pathways involving PbRpt3 in *Plasmodium*, we performed a global immunoprecipitation of PbRpt3-mCherry obtained from mixed trophozoite-schizonts soluble extracts, followed by a mass-spectrometry analysis of the protein complexes (IP/MS). Four biological replicates were analyzed via IP/MS along with the wild-type parental line used as a control. This analysis led to the identification of 2713 proteins, of which 623 were significantly enriched in the PbRpt3-mCherry IP (T test *q*-value < 0.01). (Supplementary Table S3, sheet 1). The detection of PbRpt3 bait confirmed the quality of the IP/MS. The entire putative proteasome regulatory particle ATPase Rpt (Rpt1 to 6) were identified, as well as all the regulatory particles of non-ATPase Rpn (Rpn 1 to 13) (Figure 6A). Of note, none of the alpha or beta subunits belonging to the catalytic 20S proteasome complex were present among the significantly enriched 623 proteins. A previous study reported the characterization of the 26S proteasome of *P. falciparum* using an affinity purification strategy based on the ubiquitin-like domain of PfRad23, one of the ubiquitin receptors which target ubiquitylated proteins to the proteasome [5]. In this study, all the 19S and 20S proteasome subunits were identified in a mixed population of trophozoites and early schizonts. However, *P. falciparum* parasites were subjected to formaldehyde crosslinking prior to analysis, thus allowing us to detect both stable and transient interactions. These observations, together with the absence of the *P. berghei* 20S subunits in our study, suggest that the association of 19S and 20S is transient and dynamic in the parasite.

PbRpt3 was initially identified in the PbPP1c interactome, in both schizont and gametocyte stages [14]. However, in the reverse experiment, PbPP1c is not detected among the potential partners of PbRpt3. As mentioned above, our 3D model and results in the *Xenopus* oocytes suggest that PP1c would not interact with PbRpt3 when complexed in the proteasome. Since we detected all 19S proteasome subunits in the interactome of PbRpt3, the absence of PbPP1c could be explained by the fact that PbRpt3–proteasome complexes are predominantly present in the parasite, preventing the detection of a potential PbPP1c–PbRpt3 interaction. It is also important to note that the IP was performed using parasite extracts from a mixture of trophozoite and schizont stages. These stages have been chosen based on our localization results showing PbRpt3 expression. In schizonts, PP1c has been described to be involved in controlling the initiation of egress [18]. Furthermore, transcriptomic analyses together with studies on proteasome inhibition during the parasite intraerythrocytic cycle suggest that the 26S complex, including Rpt3, is more highly expressed in late stages [48,50]. Consequently, since PP1c and PbRpt3 appear to play specific roles in late parasite stages, the PbPP1c–PbRpt3 interaction may occur earlier during the intraerythrocytic cycle.

To further gain new insights into pathways involving PbRpt3 in the parasite, we performed a Gene Ontology enrichment analysis including the 623 potential partners using PlasmoDB software (Release 63, May 2023). Enriched GO terms/pathways of computed and curated biological processes, cellular components, and molecular functions were analyzed and summarized in Supplementary Table S3, sheet 2. Ten GO terms were found to be significantly enriched, corresponding to thirty-nine different *P. berghei* proteins, including PbRpt3 (Supplementary Table S3, sheet 3). Among them, 19 proteins belong to the ubiquitin-proteasome pathway. This includes the 6 Rpt (Rpt1 to 6), as well as the 13 Rpn (Rpn 1 to 13) regulatory particles. We did not observe the presence of other proteasome-related proteins such as the two proteasome ubiquitin receptors, Rad23 and Dsk2, that were identified in the analysis performed by Wang et al. [5].

In addition to the 26S proteasome-related pathways, our GO analysis revealed a significant enrichment in the “intracellular protein-containing complex” pathway (Cellular components, GO:0140535) and “phospholipid binding pathway” (Molecular function, GO:0005543) (Supplementary Table S3, sheet 2). In order to obtain insights into the potential relation with the proteasome 19S subunit, the 39 proteins belonging to enriched pathways underwent the retrieval of networks for interacting proteins using STRING software (v.11.5, July 2023). Interestingly, this analysis uncovered seven proteins belonging to the “phospholipid binding pathway” (Figure 6B, Supplementary Table S3, sheet 3; Supplementary Figure S10). Among these proteins, we found the phosphoinositide-binding protein PH2 (PBANKA_1351400), which has been described to play a role during the fusion between the micronemes membrane and cytoplasmic membrane in *P. falciparum* [51], the phosphoinositide-specific phospholipase C (PI-PLC, PBANKA_1211900), the double C2-like domain-containing protein (DOC2, PBANKA_1457200), and the phosphoinositide-binding protein PX1 (PBANKA_0618200). Five additional phospholipases were found among the 623 potential PbRpt3 partners (Supplementary Table S3, sheet 1), supporting a possible role of PbRpt3 in the regulation of phospholipid hydrolysis. We also observed, linked to the phosphoinositide-binding protein PX1 in our STRING analysis, the calcium-dependent protein kinase 7 (CDPK7, PBANKA_0925200). Interestingly, PfCDPK7 is involved in the regulation of phospholipid biosynthesis in *P. falciparum* [52], and it has been described as a partner of PfRCC-PIP, a *Plasmodium*-specific regulator of PfPP1c [28]. This suggests that, like RCC-PIP, PbRpt3 could provide a platform to regulate phosphorylation/dephosphorylation processes, possibly linked to membrane phospholipids. Taken together, although these potential interactions should be confirmed by complementary approaches, our observations support a role for PbRpt3, within the 19S proteasome or alone, in various crucial parasite mechanisms linked to membrane dynamics.

### 2.11. Conclusions

Our study revealed an undescribed relationship between Rpt3, a component of the *Plasmodium* 19S proteasome regulatory complex, and the PP1 phosphatase. Using in vitro approaches and a heterologous model, we demonstrated the ability of PbRpt3 to activate PP1c. We observed that the up-regulation of PP1c dephosphorylation activity is not only dependent on the direct binding of PbRpt3 to PP1c via its RVxF motifs but also potentially on ATP-binding capacity of PbRpt3. In addition, analysis of the PbRpt3 interactome in the parasite confirmed that it belongs to the 19S complex and that PbRpt3–PbPP1c interaction may occur outside the proteasome complex. It also revealed the potential interaction of PbRpt3 with proteins involved in phospholipid binding. These observations, combined with the inability to generate KO parasites for PbRpt3, suggest that PbRpt3 plays a critical role in various pathways in *Plasmodium*, either within or outside the proteasome complex. This role is likely achieved through binding and activation of PP1c and/or interaction with phospholipids.

## 3. Materials and Methods

### 3.1. Ethics Statement

Mice were purchased from Charles River and housed in an Animal Biosafety Level 2 facility at the Institut Pasteur de Lille. Mature *Xenopus laevis* females were purchased from the CRB-University of Rennes I and housed in PHExMAR, University of Lille. All animals were maintained in accordance with the French National Guidelines for Use of Animals for Scientific Purposes which is also in line with EU Directive 2010/63/EU. Experimental protocols performed in this study were reviewed and approved by the “Comité d’Ethique CEEA-75 en Experimentation Animale Nord-Pas de Calais-France” (mice project number: 18905-2019020111166978v2; *Xenopus* project number: G59-00913).

### 3.2. Animals

Mice: Infections with the parasite *Plasmodium berghei* were performed in 4 weeks CD1 male mice maintained in filter cages and sorted randomly into groups of 3–4 animals. Mice were infected with parasites resuspended in phosphate buffered saline by intraperitoneal injection. Drugs were administered in drinking water or via intraperitoneal injection. Intra-erythrocytic parasitemia was monitored regularly on blood smears.

*Xenopus*: After anesthesia, realized by immersion in 2 g/L MS222 solution (tricaine methane sulfonate), ovarian lobes were surgically removed and stored in ND96 medium (96 mM NaCl, 2 mM KCl, 1.8 mM CaCl_2_, 1 mM MgCl_2_, 5 mM HEPES-NaOH, pH 7.5) at 19 °C.

### 3.3. PbRpt3 Sequence Analysis and Molecular Modeling

We used PlasmoDB data base version 63 (May 2023) as a reference for gene annotation and expression [https://plasmodb.org]. The model was constructed using the sequence alignment of *Plasmodium berghei* PbRpt3 [PBANKA_0715600] and the human PSMC4 of 418 amino acids (PDB file 6MSB—chain D). This protein shows 67% identity with PbRpt3 (Supplementary Figure S2). The crystallographic structures available on PDB (Protein Data Bank, https://www.rcsb.org/ (accessed in August 2022)) allowed us to build a 3D model of PbRpt3 by homology modeling. The magnesium ion was copied from the reference, the atomic partial charges were calculated with the Gasteiger–Hückel method, the geometry of the backbone was optimized through 2000 iteration steps of conjugated gradients, and the protein was allowed to move until the gradient value was smaller than 0.01 kcal mol^−1^ Å^−1^ with the MMFF94s force field [53]. The quality of the model was checked based on its Ramachandran plot (Supplementary Figure S11). The calculations were performed with SYBYL software version 6.9.2 (Tripos Associates, St. Louis, MO, USA), and the images were generated with Chimera software version 1.15. PbRpt3 was finally placed in the human proteasome by replacing the D chain in order to have a representation of its environment (PDB file 6MSB).

### 3.4. Plasmids and Directed Mutagenesis

Plasmids pGADT7 and pETDuet-1 were purchased from Clontech and Novagen, respectively. The plasmid pL1886 was kindly provided by Dr B. Franke-Fayard (Leiden University Medical Center, Leiden, The Netherlands). The plasmid PbGEM-022521 used in the reverse genetic study in *P. berghei* was kindly gifted from the Plasmogem-Wellcome Sanger Institute (Cambridge, UK). For *Xenopus* oocytes experiments, a PbRpt3 cDNA with optimized codons was synthetized and inserted into the pGADT7 plasmid (Genescript; Supplementary Figure S7). The plasmids pGADT7-PbRpt3-HA mutated 305-RKAEA-309 and pGADT7-PbRpt3-HA substituted for alanines in positions K188, D241, N288, and Q356, both optimized for *Xenopus laevis*, were purchased from Azenta (Supplementary Figure S7). For the other mutations on pGAD-T7-PbRpt3, site-directed mutations were performed using the NEB Q5 Hot Start High-Fidelity DNA Polymerase (New England Biolabs, Ipswich, MA, USA; cat # M0493) with primers generated via the NEBaseChanger tool (https://nebasechanger.neb.com/ (accessed in March 2022)) and listed in Supplementary Table S2. This strategy was used for mutating 200-KVTF-203 in 200-KATA-203 on pGADT7-PbRpt3 (primers KA3 and KA4), and to obtain the double mutant, the mutation 305-RKAEA-309 was performed on mutated pGADT7-PbRpt3-KATA (primers RK3 and RK4). The PCR reactions were performed as recommended by the manufacturer (New England Biolabs) with optimized annealing temperatures, as indicated in Supplementary Table S2. After analysis, 1 µL of each PCR product was treated with KLD (kinase, ligase, DpnI) enzyme mix, and 5 µL of the treated mix were used to transform competent bacteria (Takara, Saint-Germain-en-Laye, France; Stellar™ Competent Cells, cat # 636763). Mutants were checked by sequencing, and all primers used in this study are indicated in Supplementary Table S2.

### 3.5. Recombinant Protein Expression

The coding regions of PbRpt3-45 kDa (a.a. 1–395) and PbRpt3-30 kDa (a.a. 131–395) were obtained via PCR with primers P1–P2 and P2–P4, respectively (Supplementary Table S2) and cloned into pETDuet-1 (Novagen) using the In-Fusion HD Cloning system (Takara). All recombinant protein constructs were verified via sequencing. Recombinant his-tagged PbRpt3 expression was carried out in Artic Express BL21 Star™ (DE3) Chemically Competent *E. coli* cells (Life Technologies, Carlsbad, CA, USA) in the presence of 0.5 mM IPTG at 10 °C for 24 h (total culture volume: 1 L). After centrifuging at 3900 rpm for 30 min at 4 °C, cells were frozen at −80 °C overnight. Cells were resuspended in non-denaturing buffer at 4 °C (15 mL; 10 mM Tris, 500 mM NaCl, MgCl_2_ 1 mM, 3 U of DNase I, and protease inhibitor cocktail 1x from Roche, Basel, Switzerland; cat # 4693116001, pH 7.9) followed by sonication and ultracentrifugation at 13,000 rpm for 45 min at 4 °C. Pellets were resuspended in denaturing buffer (40 mL; 10 mM Tris, 500 mM NaCl, 6 M guanidine, 20 mM imidazole, MgCl_2_ 1 mM and 1x protease inhibitor, pH 7.9) and sonicated again. After ultracentrifugation at 18,000 rpm for 45 min at 4 °C, the supernatant was incubated overnight at 4 °C with Ni-NTA agarose beads (Qiagen, Courtaboeuf, France), used to purify the recombinant proteins as described [27]. The beads were centrifuged at 3900 rpm for 20 min at 4 °C and washed four times in denaturing buffer. Proteins were eluted with 12 mL of elution buffer (20 mM Tris, 500 mM NaCl, 6 M guanidine, 600 mM imidazole, 1 mM MgCl_2_ and 1x Roche protease inhibitor) and exchanged into #1 to #5 dialysis buffers in order to remove the imidazole and the guanidine. Exchange buffers were prepared with 10% glycerol deionized water, 500 mM NaCl, 10 mM TrisHCl, 1 mM MgCl_2_, pH 7.9. Exchange buffers #1, #2 and #3 additionally contained guanidine at concentrations of 6 M, 4 M, and 2 M, respectively, and exchange buffers #4 to #5 were identical and guanidine free. After exchange in buffer #4, proteins were ultracentrifuged at 13,000 rpm for 10 min at 4 °C and concentrated to 1.5 mL using centrifugal filters (Amicon Ultra-4, Merck, Molsheim, France). They were then exchanged into buffer #5 overnight at 4 °C and aliquoted and stored at −80 °C. Recombinant proteins were quantified with a Pierce™ BCA Protein Assay Kit (Life Technologies), and the purity of the purified proteins was checked using SDS-PAGE and Western blot using anti-His antibody (1:1000, Qiagen) as the primary antibody, HRP-labelled anti-mouse IgG (1:20,000 dilution, Rockland Immunochemicals, Pottstown, PA, USA) as the secondary antibody, and chemiluminescence detection (SuperSignal™ West Dura Extended Duration Substrate, Life Technologies).

### 3.6. Measurement of Binding of PbRpt3 to PP1c

PfPP1c (PF3D7_1414400) shares a high level (99%) of amino acid identity (301/304) with the PbPP1c sequence (PBANKA_1028300); therefore, we used the PfPP1c recombinant protein that we previously purified [25]. To assess binding of recombinant PbRpt3-30kDa and PbRpt3-45kDa proteins to PfPP1c, an ELISA-based assay was used as previously described [26]. Nunc-Immuno^TM^ Microwell Maxisorp plates (Merck) were coated overnight at 4 °C with 25 pmol/100 µL of either PbRpt3-30kDa, PbRpt3-45kDa, or PfI2 as a positive control, diluted in PBS. After five washes with 0.1% PBS-Tween, the plates were blocked with 200 µL/well of PBS containing 0.5% gelatin for 1 h at room temperature. Plates were then incubated at 37 °C for 2 h with different quantities (0.125–4 pmol) of biotinylated PfPP1c labeled with biotin-NHS Calbiochem^TM^, Fisher Scientific, Illkirch, France) in PBS-Tween 0.1%. The binding detection was performed using Streptavidin-HRP (1:150,000 in 0.1% PBS Tween) and trimethylbenzidine substrate (Uptima) (100 µL/well). The reaction was stopped using 2 N HCl. An ELISA plate reader (Multiskan FC, Thermo Fisher Scientific, Waltham, MA, USA) was used to measure the optical density at 450 nm and 570 nm. The difference between these two OD measures was used for analysis. BSA was used as a control. The statistical significance was calculated with the Mann–Whitney U test for nonparametric data. *p* values < 0.05 were considered significant.

### 3.7. Assays for Effects of PbRpt3 on PfPP1 Activity

To investigate the role of PbRpt3 on PfPP1c activity, p-nitrophenyl phosphate (pNPP) was used in an assay as a substrate as described previously [25]. Briefly, different quantities of PbRpt3-30-kDa or PbRpt3-45kDa (37.5 to 600 pmol) were preincubated for 30 min at 37 °C with 1 µg of PfPP1c. After adding the substrate, the plate was incubated 1 h at 37 °C, and the variation in phosphatase activity of PfPP1c in the presence of PbRpt3 was measured based on optical density at 405 nm. Results are presented as mean of increase or decrease of PP1 phosphatase activity in comparison with PfPP1c activity without PbRpt3.

### 3.8. Analysis of Xenopus Oocytes GVBD and Protein Immunoprecipitation

The five plasmids (pGADT7-PbRpt3, pGADT7-PbRpt3-KATA, pGADT7-PbRpt3-RKAEA, pGADT7-PbRpt3-KATA/RKAEA, and pGADT7-PbRpt3 ATP-binding site mutant) were linearized with XhoI and used as templates with the T7 mMessage mMachine kit (Ambion^TM^, Thermo Fisher Scientific) to synthesize in vitro capped mRNA (cRNA) encoding full-size 45 kDa native or mutated protein PbRpt3. *Xenopus* oocytes were micro-injected with 80 nL of water used as control, or with cRNA coding for PbRpt3 native/mutants (60 ng in 80 nL). We also injected PfI2 cRNA as a positive control (60 ng in 80 nL) [27]. Microinjections were followed by progesterone extracellular treatment when indicated (10 μM, 15 h after micro-injection). After 15 h, GVBD (germinal vesicle breakdown) was detected by the appearance of a white spot at the center of the animal pole [54]. Experiments were performed using ten oocytes and repeated on two to three animals. For immunoprecipitation experiments, 20 oocytes were lysed in 200 µL of buffer PY (50 mM Hepes pH 7.4, 500 mM NaCl, 0.05% SDS, 5 mM MgCl_2_, 10 µg/mL aprotinin, 10 µg/mL soybean trypsin inhibitor, 1 mg/mL bovine serum albumin, 10 µg/mL leupeptin, 1 mM PMSF, 10 µg/mL benzamidine, 1 mM sodium vanadate) and centrifuged at 4 °C for 15 min at 12,000× *g*. Supernatants were incubated with anti-XePP1c (1:200, Santa-Cruz Biotechnology, Heidelberg, Germany), anti-HA (1:200, Invitrogen, Thermo Fisher Scientific), or anti-*Xenopus* Rpn10 (PSMD4) (1:150, Santa Cruz Biotechnology) antibodies in the presence of protein A-sepharose beads (20 µL of 50% bead solution, Merck) for 1 h at 4 °C. After centrifugation, the beads were washed three times with buffer PY, and immune complexes were eluted with 25 µL of Laemmli buffer 2X (Biorad) heated at 90 °C for 5 min. For electrophoresis, 15 µL of proteins complex solution were separated on 4–20% SDS-PAGE gels (mini protean TGX, BioRad, Hercules, CA, USA), blotted onto nitrocellulose, and probed with primary antibody anti-XePP1 (Santa-Cruz Biotechnology, 1:1500), anti-HA (1:1500, Invitrogen, Waltham, MA, USA), or anti XeRpn10 (Santa-Cruz Biotechnology, 1:1500) followed by anti-mouse or anti-rabbit secondary antibodies (1:10,000, Trueblot, Rockland Immunochemicals). Chemiluminescence detection was performed with an ECL Select detection system (Amersham^TM^, Merck) on hyperfilm (MP, Amersham^TM^, Merck).

### 3.9. Isolation of Parasites Stages

Parasite stages were isolated as previously described [55,56,57]. Trophozoite stage parasites were obtained from blood collected via cardiac puncture from euthanized mice at a parasitaemia <5%. For immunofluorescence experiments, mature schizonts were obtained from blood cultured (20 h of culture) in a RPMI 1640 medium containing 25 mM HEPES, 0.4% Albumax, 0.2 mM hypoxanthine, and 20 µg/mL gentamycin. For the immunoprecipitation experiment, a mixed schizonts population (early, middle, and late schizonts) was obtained after 15 h of culture in the same medium. This mixed population was purified on a 60% Nycodenz gradient (27.6% *w*/*v* Nycodenz in 5 mM Tris-HCl pH 7.20, 3 mM KCl, 0.3 mM EDTA) and centrifuged for 20 min at 450× *g*. Each biological sample for IP-MS analysis was obtained by mixing the parasite populations from seven mice. For the selection of gametocytes, 200 µL of phenylhydrazine (6 mg/mL) were injected (IP) in mice three days prior to parasite infection. On day 3 post-infection and for two days, mice were treated with sulfadiazine (Sigma) 20 mg/L in drinking water, then, on day 5 post infection, blood collection was performed via cardiac puncture on euthanized mice. For all parasite stages, the purity of the parasite preparations was checked on Giemsa-stained smears via microscopic examination.

### 3.10. Generation of Transgenic Parasites

The *P. berghei* PbANKA-GFP parasites were a kind gift from Dr. O. Silvie (Université Pierre et Marie Curie, Paris, France), and the pG230 parasites were a kind gift from Dr. N. Philip (The University of Edinburgh, Edinburgh, UK). A PbRpt3 KO line was generated via double homologous recombination of a NotI-linearized PlasmoGEM vector (PbGEM-022521, Wellcome Sanger Institute) transfected into PbANKA-GFP parasites. The C-terminal m-Cherry-tagged PbRpt3 line was generated by single homologous recombination: a 1228-bp region of PbRpt3 starting 289 bp downstream from the start codon and lacking a stop codon was inserted into pL1886 vector (Genescript). A silent mutation was inserted in the coding sequence to generate an AvrII single cutter restriction site (Figure 5) in order to linearize the plasmid before transfection into PbANKA-GFP strain. All transfections were performed as previously described [45,58,59]. Nycodenz-enriched schizonts were electroporated with 10 µg of linearized DNA and IV-injected into naive mice. Positive transfectants were selected with pyrimethamine in drinking water (70 mg/L, TCI). Four independent transfections were performed to generate KO lines, with a total number of six recipient mice. KO transgenic parasites were cloned by limiting dilution (10 mice per cloning experiment) and genotyped using diagnostic PCR (Supplementary Table S2, Supplementary Figure S9). Primers QCR2—GW2 and GW1—GT were used to detect the integration of the dhfr resistance cassette at the PbRpt3 locus (5′- and 3′ side respectively), and primers QCR2—P1/QCR2—P4 and/or QCR3—P4 were used to detect the wild-type PbRpt3 gene. PbRpt3-mCherry parasites were enriched via cell-sorting on a FACSAria cell sorter (Beckton Dickinson, Franklin Lakes, NJ, USA), and mCherry integration was confirmed by PCR using primers PF-877 (Supplementary Table S2). The correct size expression of PbRpt3-mCherry protein was confirmed by Western blot analysis, with the parental strain used as a control. Samples in Laemmli buffer were denatured at 100 °C for 3 min and electrophoresed on a 4–20% SDS polyacrylamide gel. Proteins were transferred onto nitrocellulose membranes (Amersham^TM^, Merck). Membranes were probed with rabbit anti-RFP pAb (1:2000, MBL, PM005) as the primary antibody and anti-rabbit HRP (1:20,000, Sigma-Aldrich, St. Louis, MO, USA, A0545) as the secondary antibody. Chemiluminescence detection was performed as above.

### 3.11. Immunofluorescence Assays

Blood was collected from mice infected with *P. berghei* PbRpt3-mCherry parasites (total number of mice used for IFA: 6). *P. berghei* blood stages were fixed with 4% paraformaldehyde and 0.075% glutaraldehyde for 10 min at 4 °C and centrifuged at 2000 rpm 2 min at room temperature. Sedimentation, permeabilization, and saturation steps were performed as described [13]. Then, a 1:500 dilution in PBS BSA 1% of Anti-RFP pAb (MBL, PM005) was applied for 1 h at 37 °C. Following PBS washing, coverslips were incubated with anti-Rabbit IgG Cross-Adsorbed, Alexa Fluor 594 (Life Technologies, A11012) diluted at 1:1000 in PBS BSA 1%, and parasitic nuclei were stained with DAPI at a concentration of 1 μg/mL in PBS BSA 1% for 1 h at 37 °C. The coverslips were mounted in Mowiol (3 µL), and confocal imaging was performed using a Zeiss LSM880 microscope (Zeiss, Oberkochen, Germany) Subsequent image treatment was performed with ImageJ software (https://imagej.net/ij/).

### 3.12. Immunoprecipitation

Purified parasites (mixed schizont population) of parental wild-type parasites used as control and PbRpt3-mCherry were suspended with 50 mM Tris-HCl, 0.5% Triton X100, and protease inhibitor cocktail (Roche), pH 8. A total of 10 freeze-thaw cycles and sonication 30″on/off cycles followed by a 5 h centrifugation at 13,000 rpm at 4 °C allowed us to obtain the soluble fractions. RFP-Trap1_A beads (Chromotek, Planegg-Martinsried, Germany) were equilibrated with dilution buffer (20 mM Tris, 150 mM NaCl, 0.5% Triton X-100 and protease inhibitor cocktail (Roche), pH 7.5) and incubated overnight at 4 °C with parasite soluble extracts on a rotating wheel. Beads were washed 10 times with dilution buffer, and elution was performed in Laemmli buffer. After 3 min at 100 °C, eight samples were stored at −20 °C for subsequent mass spectrometry experiments (four biological samples for schizonts (15 h culture) PbWT-GFP and four biological samples for schizonts (15 h culture) PbRpt3-mCherry). The presence of PbRpt3-mCherry in the IP was checked via Western blot analysis as above.

### 3.13. Sample Preparation for Mass Spectrometry

S-Trap^TM^ micro spin column (Protifi, Hutington, NY, USA) digestion was performed on immunoprecipitation eluates according to the manufacturer’s instructions. Briefly, samples were supplemented with 20% SDS to a final concentration of 5%, reduced with 20 mM TCEP (Tris(2-carboxyethyl) phosphine hydrochloride), and alkylated with 50 mM CAA (chloracetamide) for 5 min at 95 °C. Aqueous phosphoric acid was then added to a final concentration of 2.5% followed by the addition of S-Trap binding buffer (90% aqueous methanol, 100 mM TEAB, pH 7.1). Mixtures were then loaded on S-Trap columns. Five washes were performed for thorough SDS elimination. Samples were digested with 3 µg of trypsin (Promega, Madison, WI, USA) at 47 °C for 2 h. After elution, peptides were vacuum dried.

### 3.14. NanoLC-MS/MS Protein Identification and Quantification

The tryptic peptides were resuspended in 35 µL, and a volume of 3 µL was injected in a nanoElute (Bruker Daltonics, Bremen, Germany) HPLC (high-performance liquid chromatography) system coupled to a timsTOF Pro (Bruker Daltonics) mass spectrometer. HPLC separation (Solvent A: 0.1% formic acid in water; Solvent B: 0.1% formic acid in acetonitrile) was carried out at 400 nL/min using a packed emitter column (C18, 25 cm × 75 μm 1.6 μm) (Ion Optics, Fitzroy, Australia) using a 15 min gradient elution (2 to 17% solvent B for 8 min; 17 to 25% for 2 min; 25% to 37% for 2 min; 37% to 95% for 1 min, and finally, 95% for 2 min to wash the column). Mass-spectrometric data were acquired using the parallel accumulation serial fragmentation (PASEF) acquisition method in DIA (Data Independent Analysis) mode. The measurements were carried out over the *m*/*z* range from 475 to 1000 Th. The range of ion mobility values was from 0.85 to 1.30 V s/cm^2^ (1/k0). The total cycle time was set to 0.95 s.

Data analysis was performed using DIA-NN software (version 1.8.1) [60]. A search against the UniProt/Swiss-Prot *Mus Musculus* database downloaded from Uniprot on 09/01/2023 (17,574 entries) and *P. berghei* ANKA database downloaded from PlasmoDB on 17/05/2023 (Release 63) was performed using library free workflow. For this purpose, “FASTA digest for library free search/library generation” and “Deep learning spectra, RTs and IMs prediction” options were checked for precursor ion generation. A maximum of 1 trypsin missed cleavage was allowed, and the maximum variable modification was set to 2. Carbamidomethylation (Cys) was set as the fixed modification, whereas protein N-terminal methionine excision, methionine oxidation, and N-terminal acetylation were set as variable modifications. The peptide length range was set to 7–30 amino acids, precursor charge range 2–4, precursor m/z range 300–1800, and fragment ion m/z range 300–1800. To search the parent mass and fragment ions, accuracy was set to 10 ppm manually. The false discovery rates (FDRs) at the protein and peptide level were set to 1%. Match between runs was allowed. For the quantification strategy, Robust LC (high precision) was used as advised in the software documentation, and the normalization option was disabled, whereas default settings were kept for the other algorithm parameters.

Statistical and bioinformatic analysis including volcano plot with Perseus were performed with Perseus software (version 1.6.15) [61], freely available at www.perseus-framework.org (accessed in 20 May 2023). All protein intensities were log2 transformed to perform statistics. For statistical comparison, we set two groups (WT, RPT3ch), each containing up to four biological replicates. We then filtered the data to keep only proteins with at least four out of four valid values in at least one group. Next, the data were imputed to fill missing data points by creating a Gaussian distribution of random numbers with a standard deviation of 33% relative to the standard deviation of the measured values and 2.5 standard deviation downshift of the mean to simulate the distribution of low signal values. We performed a t-test, FDR < 0.01, S0 = 2.

## Data Availability

The mass spectrometry proteomics data have been deposited to the ProteomeXchange Consortium via the PRIDE [62] partner repository with the dataset identifier PXD044616.

## Supplementary Materials

The following supporting information can be downloaded at: https://www.mdpi.com/article/10.3390/ijms262311720/s1.
