# Supplementary material for: Characterization of the Regulatory AAA-ATPase Subunit Rpt3 in Plasmodium berghei as an Activator of Protein Phosphatase 1
Source: Int J Mol Sci. 2025 Dec 3;26(23):11720. doi: 10.3390/ijms262311720 (PMC12691818; doi:10.3390/ijms262311720)
Supplement: Supplementary file 1 [file ijms-26-11720-s001.zip › Supplementary Figures.pdf]

Supplementary Materials

Characterization of the regulatory AAA-ATPase subunit Rpt3 in *Plasmodium berghei* as an activator of Protein Phosphatase 1

Claudianne Lainé[1a], Caroline De Witte[1a], Alain Martoriati[2], Amaury Farce[3], Inès Metatla[4], Ida Chiara Guerrera[4], Katia Cailliau[2], Jamal Khalife[1], Christine Pierrot[1*]

1. Univ. Lille, CNRS, Inserm, CHU Lille, Institut Pasteur de Lille, U1019 - UMR 9017 -CIIL - Center for Infection and Immunity of Lille, F-59000 Lille, France
2. Univ. Lille, CNRS, UMR 8576-UGSF-Unité de Glycobiologie Structurale et Fonctionnelle, F-59000 Lille, France
3. University of Lille, Inserm, CHU Lille, U1286 - Infinite - Institute for Translational Research in Inflammation, F-59000 Lille, France
4. Proteomics platform 3P5-Necker, Université Paris Descartes - Structure Fédérative de Recherche Necker, INSERM US24/CNRS UMS3633, Paris, France

[a.] These authors have contributed equally to this work
[*] Corresponding author : christine.pierrot@pasteur-lille.fr

SUPPLEMENTARY FIGURE 1. Protein sequence of PbRpt3 (PBANKA_0715600)
SUPPLEMENTARY FIGURE 2. Sequence alignment of human PSMC4 with *Plasmodium berghei* PbRpt3.
SUPPLEMENTARY FIGURE 3. Superimposition of the PbRpt3 model with experimental structures chosen from the RCSB PDB.
SUPPLEMENTARY FIGURE 4. Alignment of amino acid sequence of PbRpt3 with sequences of proteins used to assess the quality of our homology model.
SUPPLEMENTARY FIGURE 5. Positioning of the RVxF motifs in the PbRpt3 homology model outside (A) and inside (B) the 26S proteasome complex.
SUPPLEMENTARY FIGURE 6. Analysis of PbRpt3 recombinant proteins.
SUPPLEMENTARY FIGURE 7. Recodonized sequence of PbRpt3 cDNA optimized for translation in *Xenopus laevis*, and deduced amino acid sequence.
SUPPLEMENTARY FIGURE 8. PbRpt3 functional analysis using *Xenopus* oocytes model.
SUPPLEMENTARY FIGURE 9. PlasmoGEM construct (PbGEM-022521) and genotyping.
SUPPLEMENTARY FIGURE 10. STRING network visualization of PbRpt3-interacting proteins belonging to the 10 enriched GO terms.
SUPPLEMENTARY FIGURE 11. Ramachandran plot and statistics of the PbRpt3 model.
References for Supplementary figures.

```
MRKMENVSKYLKEEDYYIKLKILKKQLDILNIQEEYIKEEHKNLKRELIRSKNEIKRIQS    60
VPLIIGQFLDIIDNNYGIVSSTAGSNYYVRILSTLNKEDLKPSVSVALHRHSHSIVNILP   120
SESDSSIQLLQVSERPNVKYTDLGGLDTQKQEMREAVELPLKSPELYEKIGIEPPMGILI   180
YGPPGTGKTMLVKAVANETKVTFIGVVGSEFVQKYLGEGPRMVRDVFRLARENSPSIIFI   240
DEVDAIATKRFDAQTGADREVQRILLELLNQMDGFDKSTNVKVIMATNRADTLDPALLRP   300
GRLDRKIEFPLPDRKQKRLIFQTIISKMNISSDVNIENFVVRTDKISAADIAAIAQESGM   360
QAIRKNRYIITASDFEQGYRTHVRKQLRDYEFYNI                            395
```

SUPPLEMENTARY FIGURE 1. Protein sequence of PbRpt3 (PBANKA_0715600). Two RVxF motifs are indicated in blue, the Walker A and Walker B motifs are highlighted in yellow and the sensor-1 motif is highlighted in green.

```
Percent Identity  Matrix - created by Clustal2.1


  1: HumanPSMC4_P43686_1     100.00    67.01
  2: PBANKA_0715600_PbRpt3    67.01   100.00
```

```
CLUSTAL O(1.2.4) multiple sequence alignment


HumanPSMC4_P43686_1       MEEIGILVEKAQDEIPALSVSRPQTGLSFLGPEPEDLEDLYSRYKKLQQELEFLEVQEEY 60
PBANKA_0715600_PbRpt3     --------------------MRKMENV----SKYLKEEDYYIKLKILKKQLDILNIQEEY 36
                                              *    .:     :  . ** * : * *:::*::*::****

HumanPSMC4_P43686_1       IKDEQKNLKKEFLHAQEEVKRIQSIPLVIGQFLEAVDQNTAIVGSTTGSNYYVRILSTID 120
PBANKA_0715600_PbRpt3     IKEEHKNLKRELIRSKNEIKRIQSVPLIIGQFLDIIDNNYGIVSSTAGSNYYVRILSTLN 96
                          **:*:****:*::::::*:*****:**:*****:  :*:*  .**.**:***********::

HumanPSMC4_P43686_1       RELLKPNASVALHKHSNALVDVLPPEADSSIMMLTSDQKPDVMYADIGGMDIQKQEVREA 180
PBANKA_0715600_PbRpt3     KEDLKPSVSVALHRHSHSIVNILPSESDSSIQLLQVSERPNVKYTDLGGLDTQKQEMREA 156
                          :* ***..*****:**:::*::** *:**** :*   .::*:* *:*:**:* ****:***

HumanPSMC4_P43686_1       VELPLTHFELYKQIGIDPPRGVLMYGPPGCGKTMLAKAVAHHTTAAFIRVVGSEFVQKYL 240
PBANKA_0715600_PbRpt3     VELPLKSPELYEKIGIEPPMGILIYGPPGTGKTMLVKAVANETKVTFIGVVGSEFVQKYL 216
                          *****.   ***::***:** *:*:***** *****.****:.*..:** ***********

HumanPSMC4_P43686_1       GEGPRMVRDVFRLAKENAPAIIFIDEIDAIATKRFDAQTGADREVQRILLELLNQMDGFD 300
PBANKA_0715600_PbRpt3     GEGPRMVRDVFRLARENSPSIIFIDEVDAIATKRFDAQTGADREVQRILLELLNQMDGFD 276
                          **************:**:*:******:**********************************

HumanPSMC4_P43686_1       QNVNVKVIMATNRADTLDPALLRPGRLDRKIEFPLPDRRQKRLIFSTITSKMNLSEEVDL 360
PBANKA_0715600_PbRpt3     KSTNVKVIMATNRADTLDPALLRPGRLDRKIEFPLPDRKQKRLIFQTIISKMNISSDVNI 336
                          :..*************************************:******.** ****:*.:*::

HumanPSMC4_P43686_1       EDYVARPDKISGADINSICQESGMLAVRENRYIVLAKDFEKAYKTVIKKDEQEHEFYK-  418
PBANKA_0715600_PbRpt3     ENFVVRTDKISAADIAAIAQESGMQAIRKNRYIITASDFEQGYRTHVRKQLRDYEFYNI  395
                          *::*.* ****.*** :*.***** *:*:****: *.***:.*:* ::*: :::***:
```

SUPPLEMENTARY FIGURE 2. Sequence alignment of human PSMC4 (Uniprot reference P43686-1) with *Plasmodium berghei* PbRpt3 (PlasmoDB reference: PBANKA_0715600). Sequence alignment was performed using Clustal. An asterisk indicates positions which have a single, fully conserved residue. A double dot indicates conservation between groups of strongly similar properties (scoring > 0.5 in the Gonnet PAM 250 matrix), and a single dot indicates conservation between groups of weakly similar properties (scoring =< 0.5 and > 0 in the Gonnet PAM 250 matrix).

Lainé et al, Supplementary Materials

SUPPLEMENTARY FIGURE 3. legend on next page

(A)

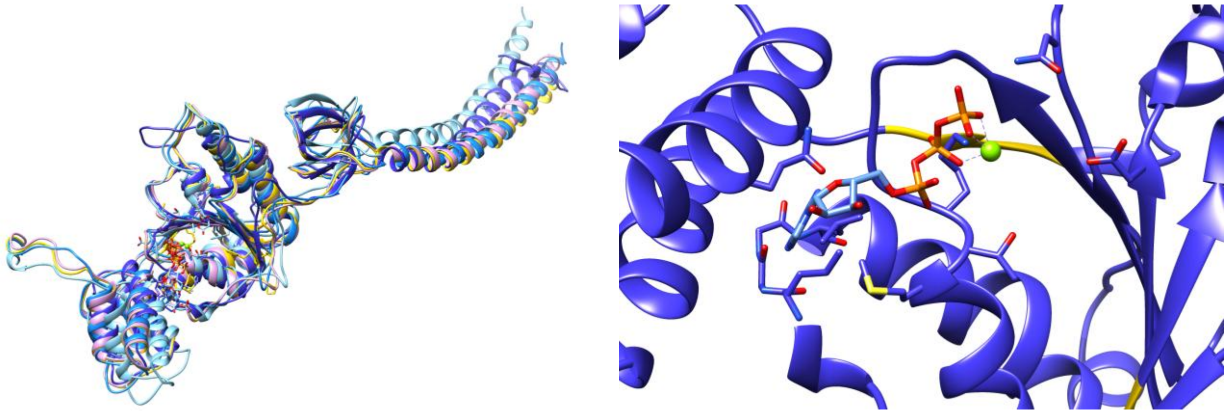

(B)

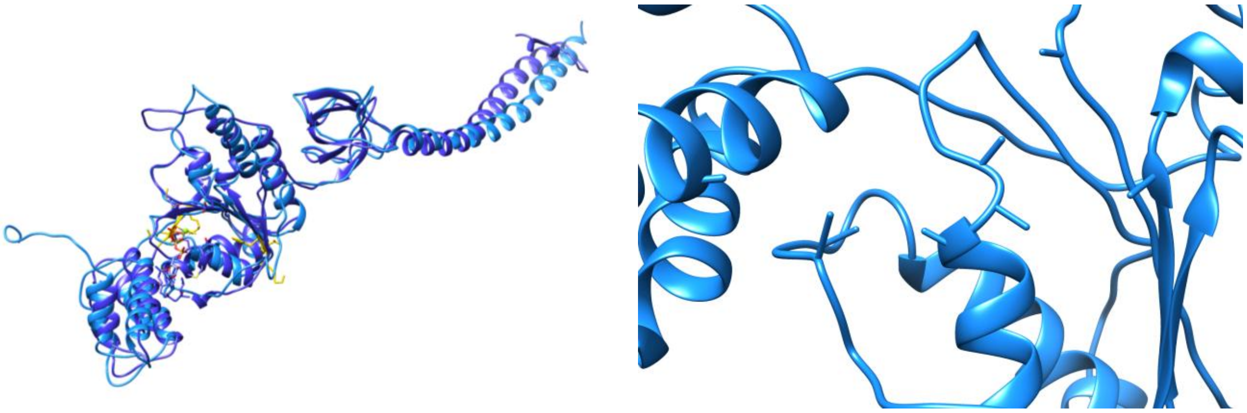

(C)

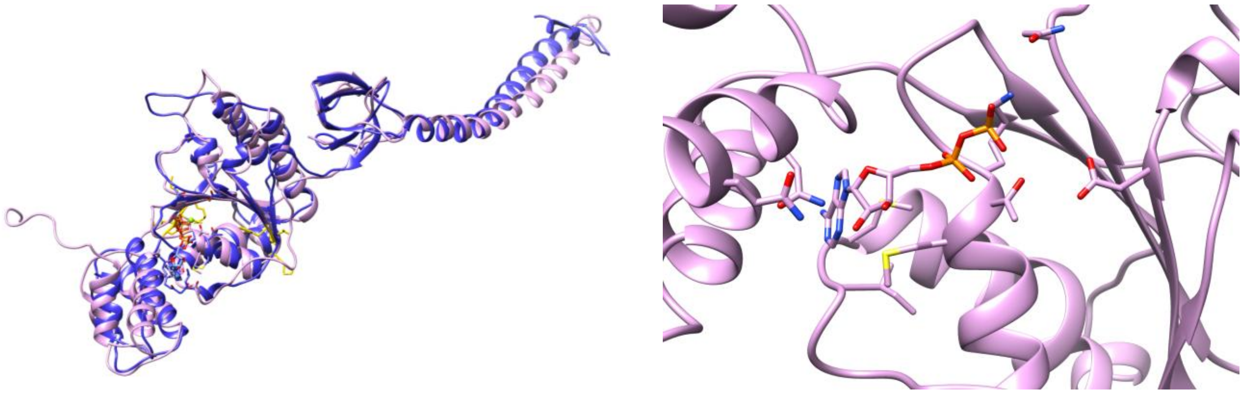

(D)

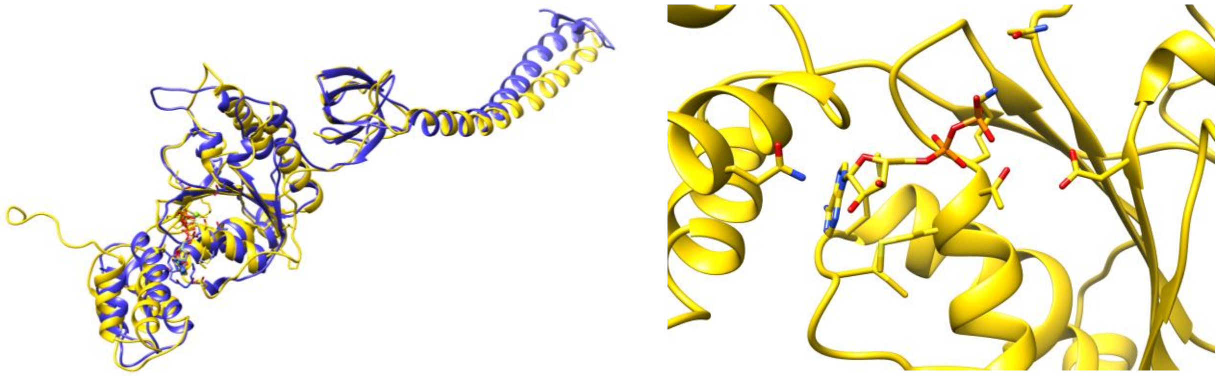

(E)

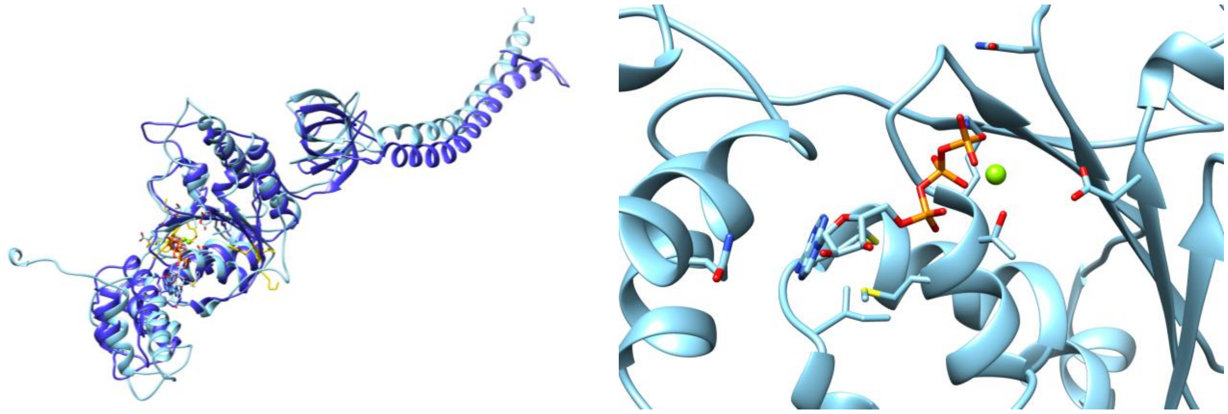

SUPPLEMENTARY FIGURE 3 (Figure on previous page): **Superimposition of the PbRpt3 model** (dark blue) **with experimental structures chosen from the RCSB PDB due to their sequence similarity with PbRpt3.** (A) all the experimental structures used, (B) 8amz (blue, [1]), (C) 5gjr (pink, [2]), (D) 5gjq (yellow, [2]), (E) 6msb (light blue, [3]). The panels on the right show closer views of the residues involved in ATP and Mg^{2+} binding. The high conservation of their position in all the structures (see Supplementary Figure 4), including our homology model, is in agreement with Hänzelmann and Schindelin [4].

```
PDB_PbRpt3.pdb_(#5)_chain_A/1-395    ..........................MRKMENVSKYLKEEDYYIKLKILK 24
6msb.cif/39-418                      ........................................DLYSRYKKLQ 10
5gjr-xa(y).pdb/39-418                ........................................DLYSRYKKLQ 10
5gjq-T(K).pdb/1-418                  ..MEEIGILVEKAQDEIPALSVSRPQTGLSFLGPEPEDLEDLYSRYKKLQ 48
8amz-D(K).pdb/1-420                  MAASAMVLDPKPVPPPPQPLEPPATVSFTASEDTSVVEEEDLYGRLKSLE 50
                                                                             * * : * *:

PDB_PbRpt3.pdb_(#5)_chain_A/1-395    KQLDILNIQEEYIKEEHKNLKRELIRSKNEIKRIQSVPLIIGQFLDIIDN 74
6msb.cif/39-418                      QELEFLEVQEEYIKDEQKNLKKEFLHAQEEVKRIQSIPLVIGQFLEAVDQ 60
5gjr-xa(y).pdb/39-418                QELEFLEVQEEYIKDEQKNLKKEFLHAQEEVKRIQSIPLVIGQFLEAVDQ 60
5gjq-T(K).pdb/1-418                  QELEFLEVQEEYIKDEQKNLKKEFLHAQEEVKRIQSIPLVIGQFLEAVDQ 98
8amz-D(K).pdb/1-420                  RQIEFKGIQEEYVKDELKNLKREHLRAQEEVKRIQSVPLVIGQFMEMVDQ 100
                                     :::::  :****:*:* ****:* :::::*:*****:**:****:: :*:

PDB_PbRpt3.pdb_(#5)_chain_A/1-395    NYGIVSSTAGSNYYVRILSTLNKEDLKPSVSVALHRHSHSIVNILPSESD 124
6msb.cif/39-418                      NTAIVGSTTGSNYYVRILSTIDRELLKPNASVALHKHSNALVDVLPPEAD 110
5gjr-xa(y).pdb/39-418                NTAIVGSTTGSNYYVRILSTIDRELLKPNASVALHKHSNALVDVLPPEAD 110
5gjq-T(K).pdb/1-418                  NTAIVGSTTGSNYYVRILSTIDRELLKPNASVALHKHSNALVDVLPPEAD 148
8amz-D(K).pdb/1-420                  NNGIVGSTTGSNYYVRILSTINRELLKPSASVALHRHSNALVDVLPPEAD 150
                                     * .**.**:***********:::* ***..*****:**:::*::**.*:*

PDB_PbRpt3.pdb_(#5)_chain_A/1-395    SSIQLLQVSERPNVKYTDLGGLDTQKQEMREAVELPLKSPELYEKIGIEP 174
6msb.cif/39-418                      SSIMMLTSDQKPDVMYADIGGMDIQKQEVREAVELPLTHFELYKQIGIDP 160
5gjr-xa(y).pdb/39-418                SSIMMLTSDQKPDVMYADIGGMDIQKQEVREAVELPLTHFELYKQIGIDP 160
5gjq-T(K).pdb/1-418                  SSIMMLTSDQKPDVMYADIGGMDIQKQEVREAVELPLTHFELYKQIGIDP 198
8amz-D(K).pdb/1-420                  SSISLLSQSEKPDVSYNDIGGCDIQKQEIREAVELPLTHHDLYKQIGIDP 200
                                     *** :*  .::*:* * *:** * ****:********.  :**::***:*

PDB_PbRpt3.pdb_(#5)_chain_A/1-395    PMGILIYGPPGTGKTMLVKAVANETKVTFIGVVGSEFVQKYLGEGPRMVR 224
6msb.cif/39-418                      PRGVLMYGPPGCGKTMLAKAVAHHTTAAFIRVVGSEFVQKYLGEGPRMVR 210
5gjr-xa(y).pdb/39-418                PRGVLMYGPPGCGKTMLAKAVAHHTTAAFIRVVGSEFVQKYLGEGPRMVR 210
5gjq-T(K).pdb/1-418                  PRGVLMYGPPGCGKTMLAKAVAHHTTAAFIRVVGSEFVQKYLGEGPRMVR 248
8amz-D(K).pdb/1-420                  PRGVLLYGPPGTGKTMLAKAVANHTTAAFIRVVGSEFVQKYLGEGPRMVR 250
                                     * *:*:***** *****.****:.*..:** *******************
```

Continued on next page

```
PDB_PbRpt3.pdb_(#5)_chain_A/1-395      DVFRLARENSPSIIFIDEVDAIATKRFDAQTGADREVQRILLELLNQMDG 274
6msb.cif/39-418                        DVFRLAKENAPAIIFIDEIDAIATKRFDAQTGADREVQRILLELLNQMDG 260
5gjr-xa(y).pdb/39-418                  DVFRLAKENAPAIIFIDEIDAIATKRFDAQTGADREVQRILLELLNQMDG 260
5gjq-T(K).pdb/1-418                    DVFRLAKENAPAIIFIDEIDAIATKRFDAQTGADREVQRILLELLNQMDG 298
8amz-D(K).pdb/1-420                    DVFRLAKENAPAIIFIDEVDAIATARFDAQTGADREVQRILMELLNQMDG 300
                                       ******:**:*:******:***** ****************:********

PDB_PbRpt3.pdb_(#5)_chain_A/1-395      FDKSTNVKVIMATNRADTLDPALLRPGRLDRKIEFPLPDRKQKRLIFQTI 324
6msb.cif/39-418                        FDQNVNVKVIMATNRADTLDPALLRPGRLDRKIEFPLPDRRQKRLIFSTI 310
5gjr-xa(y).pdb/39-418                  FDQNVNVKVIMATNRADTLDPALLRPGRLDRKIEFPLPDRRQKRLIFSTI 310
5gjq-T(K).pdb/1-418                    FDQNVNVKVIMATNRADTLDPALLRPGRLDRKIEFPLPDRRQKRLIFSTI 348
8amz-D(K).pdb/1-420                    FDQTVNVKVIMATNRADTLDPALLRPGRLDRKIEFPLPDRRQKRLVFQVC 350
                                       **:..***********************************:****:*..

PDB_PbRpt3.pdb_(#5)_chain_A/1-395      ISKMNISSDVNIENFVVRTDKISAADIAAIAQESGMQAIRKNRYIITASD 374
6msb.cif/39-418                        TSKMNLSEEVDLEDYVARPDKISGADINSICQESGMLAVRENRYIVLAKD 360
5gjr-xa(y).pdb/39-418                  TSKMNLSEEVDLEDYVARPDKISGADINSICQESGMLAVRENRYIVLAKD 360
5gjq-T(K).pdb/1-418                    TSKMNLSEEVDLEDYVARPDKISGADINSICQESGMLAVRENRYIVLAKD 398
8amz-D(K).pdb/1-420                    TAKMNLSDEVDLEDYVSRPDKISAAEITAICQEAGMHAVRKNRYVILPKD 400
                                        :***:*.:*::*::* *.****.*:* :*.**:** *:*:***:: ..*

PDB_PbRpt3.pdb_(#5)_chain_A/1-395      FEQGYRTHVR..KQLRDYEFYNI 395
6msb.cif/39-418                        FEKAYKTVIKKDEQ..EHEFYK. 380
5gjr-xa(y).pdb/39-418                  FEKAYKTVIKKDEQ..EHEFYK. 380
5gjq-T(K).pdb/1-418                    FEKAYKTVIKKDEQ..EHEFYK. 418
8amz-D(K).pdb/1-420                    FEKGYRSNVKKPDT..DFDFYK. 420
                                       **:.*:: ::  .   :.:**:
```

SUPPLEMENTARY FIGURE 4. Alignment of amino acid sequence of PbRpt3 with sequences of proteins used to assess the quality of our homology model. The residues showed in Supplementary Figure 3 are in red.

(A)

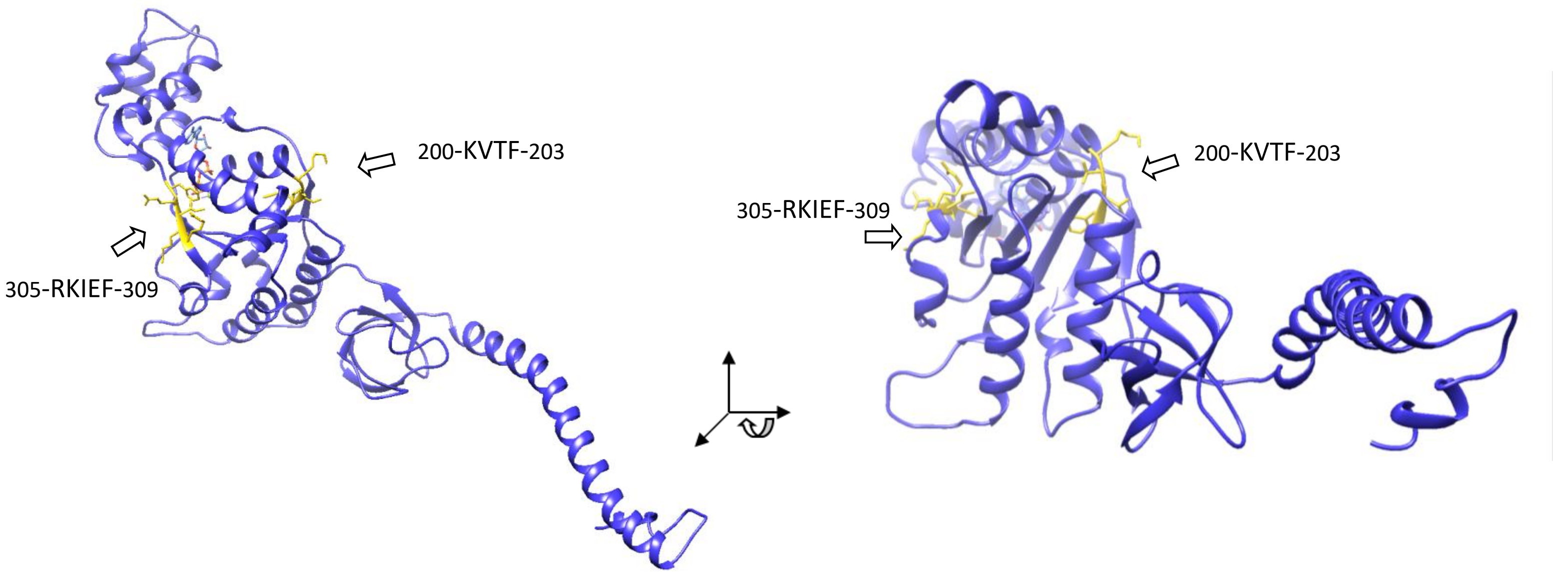

(B)

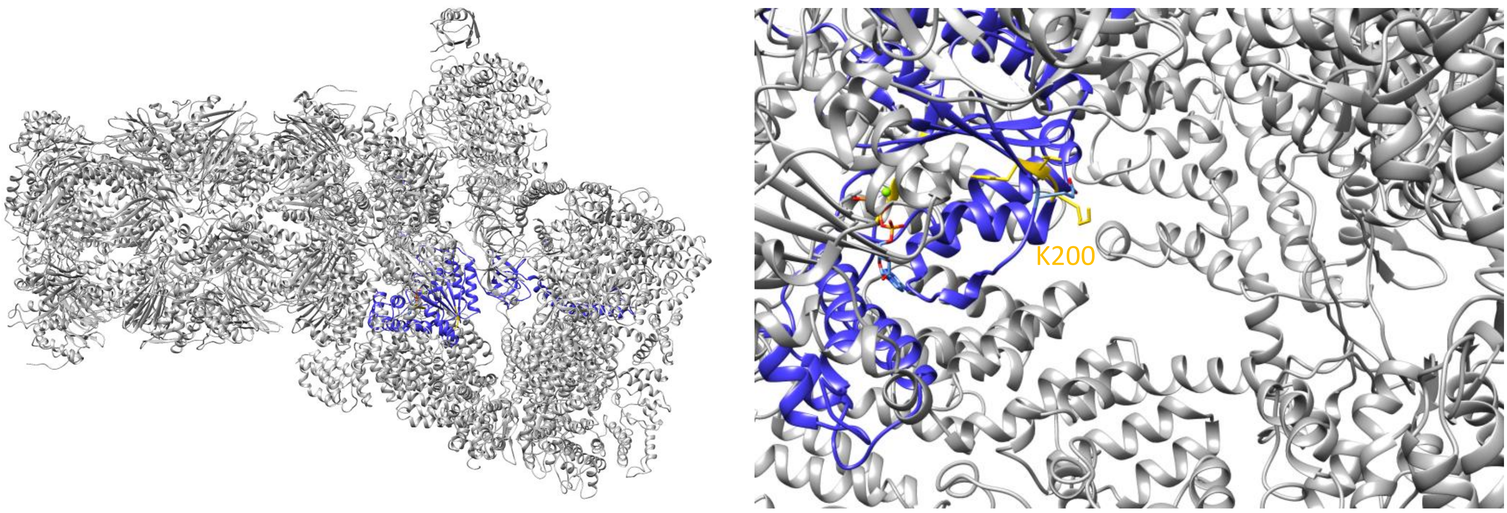

SUPPLEMENTARY FIGURE 5. Positioning of the RVxF motifs in the PbRpt3 homology model outside (A) and inside (B) the 26S proteasome complex. The RVxF motifs are in yellow. B, left panel, reconstruction of the proteasome 26S from 6msb [3] with the PMSC4 unit replaced with our PbRpt3 model, in medium blue. B, right panel, a closer view of the KVTF motif. Lysine at position 200 is in interaction with the nearby Glutamic acid 134. A UCFS chimera of the reconstructed proteasome is at the disposal of the readers to provide a better view of the opening, potentially allowing an accessibility to KVTF and the complete inaccessibility of RKIEF:
https://zenodo.org/records/17100069?token=eyJhbGciOiJIUzUxMiJ9.eyJpZCI6ImE1NjhkYzM2LTEzZjEtNDliYy04ZDdmLWM4NmMzMGFlZmY3YSIsImRhdGEiOnt9LCJyYW5kb20iOiJiMTgzYWVhOTY1YzcyZWJmNjEyNWZkOGZjZThkYjMxYiJ9.CkYIf2kUCNsfe-Blk-ER_QYR1NgXKw--7d648Q10xRXHvSX7DnP1xKtWfGseqx9CSeE-f-es87jteZFUw1J8Yg

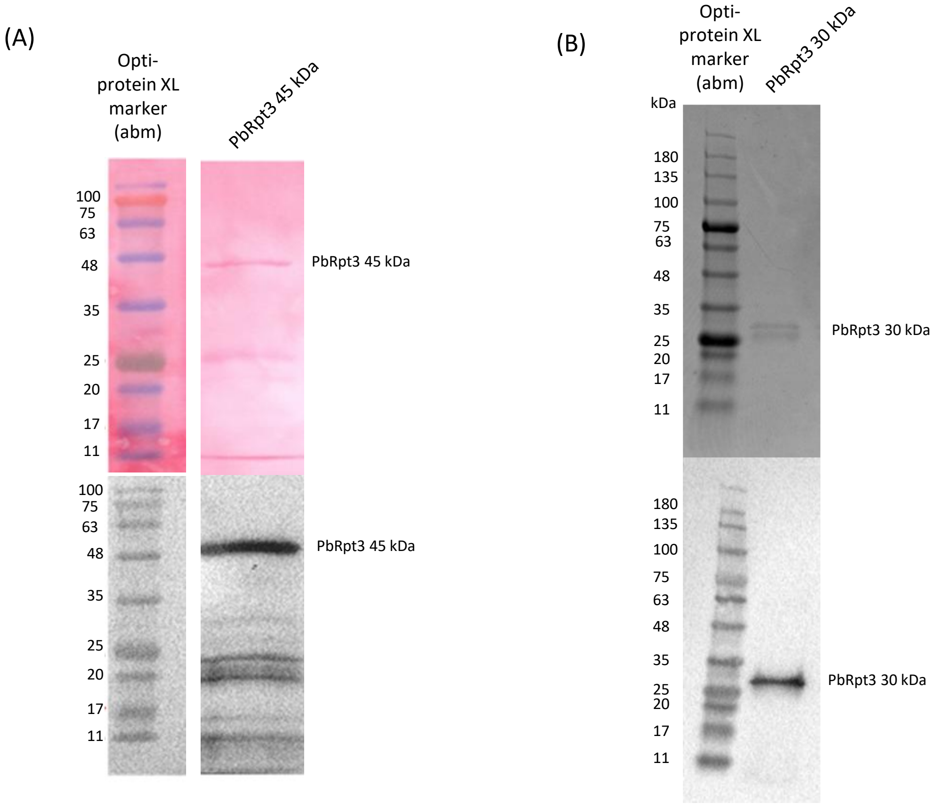

SUPPLEMENTARY FIGURE 6. Analysis of PbRpt3-45kDa (A) and PbRpt3-30kDa (B) recombinant proteins. The quality of PbRpt3-45kDa and PbRpt3-30kDa was checked by SDS-PAGE followed by ponceau or coomassie blue staining respectively (upper panel), and immunoblot analysis using an anti-His mAb (lower panel).

```
atg aga aaa atg gaa aac gtg agc aaa tac ttg aag gaa gag gac tac tac ata aaa ctg
 M   R   K   M   E   N   V   S   K   Y   L   K   E   E   D   Y   Y   I   K   L    20

aag ata ctg aag aaa caa ctt gac atc ctg aat att cag gaa gag tat atc aaa gaa gag
 K   I   L   K   K   Q   L   D   I   L   N   I   Q   E   E   Y   I   K   E   E    40

cac aaa aat ctg aag agg gag ctg atc aga tct aag aac gaa atc aag aga att cag agc
 H   K   N   L   K   R   E   L   I   R   S   K   N   E   I   K   R   I   Q   S    60

gtg cca ctg att atc gga cag ttc ctg gat atc atc gat aac aac tac gga atc gtg tct
 V   P   L   I   I   G   Q   F   L   D   I   I   D   N   N   Y   G   I   V   S    80

agc aca gcc gga tct aat tat tac gtg aga att ctg agt act ctg aac aaa gag gat ctg
 S   T   A   G   S   N   Y   Y   V   R   I   L   S   T   L   N   K   E   D   L    100

aag cct tcc gtg agt gtg gct ctg cat aga cac tct cat agc att gtg aat atc ctg cca
 K   P   S   V   S   V   A   L   H   R   H   S   H   S   I   V   N   I   L   P    120

tcc gag agt gat tcc agt att cag ctg ctg cag gtg tct gaa agg cct aac gtg aaa tat
 S   E   S   D   S   S   I   Q   L   L   Q   V   S   E   R   P   N   V   K   Y    140

aca gat ctg gga gga ctg gat act cag aag cag gaa atg aga gag gct gtg gaa ctg cca
 T   D   L   G   G   L   D   T   Q   K   Q   E   M   R   E   A   V   E   L   P    160

ctg aaa agc cct gag ctg tat gaa aag att gga atc gag cca cct atg gga att ctg atc
 L   K   S   P   E   L   Y   E   K   I   G   I   E   P   P   M   G   I   L   I    180

tac gga cca cct gga aca gga aaa act atg ctg gtg aag gca gtg gcc aat gaa aca aaa
 Y   G   P   P   G   T   G   K   T   M   L   V   K   A   V   A   N   E   T   K    200

gtg act ttt att gga gtg gtg gga tct gag ttc gtg cag aag tac ctg gga gaa gga cca
 V   T   F   I   G   V   V   G   S   E   F   V   Q   K   Y   L   G   E   G   P    220

aga atg gtt agg gat gtg ttt aga ctg gca agg gag aac tct cct agc att atc ttc att
 R   M   V   R   D   V   F   R   L   A   R   E   N   S   P   S   I   I   F   I    240

gat gaa gtg gat gct atc gca aca aaa agg ttt gat gcc cag act gga gct gat agg gag
 D   E   V   D   A   I   A   T   K   R   F   D   A   Q   T   G   A   D   R   E    260

gtg cag aga att ctg ctg gaa ctg ctg aac cag atg gat gga ttt gat aaa agc aca aat
 V   Q   R   I   L   L   E   L   L   N   Q   M   D   G   F   D   K   S   T   N    280

gtg aag gtg atc atg gca aca aac aga gcc gat act ctg gac cca gcc ctg ctg agg cca
 V   K   V   I   M   A   T   N   R   A   D   T   L   D   P   A   L   L   R   P    300

gga agg ctg gat aga aag att gag ttc cca ctg cct gat agg aaa cag aag aga ctg atc
 G   R   L   D   R   K   I   E   F   P   L   P   D   R   K   Q   K   R   L   I    320

ttc cag aca atc atc tcc aag atg aac atc tct agc gat gtg aac atc gaa aac ttc gtt
 F   Q   T   I   I   S   K   M   N   I   S   S   D   V   N   I   E   N   F   V    340

gtg aga act gat aag att tcc gct gca gat att gcc gct atc gct cag gag agt gga atg
 V   R   T   D   K   I   S   A   A   D   I   A   A   I   A   Q   E   S   G   M    360

cag gca atc agg aag aac aga tac atc atc act gca agt gat ttc gaa caa ggt tac agg
 Q   A   I   R   K   N   R   Y   I   I   T   A   S   D   F   E   Q   G   Y   R    380

aca cac gtt agg aag caa ctt aga gac tac gaa ttt tac aac att tga
 T   H   V   R   K   Q   L   R   D   Y   E   F   Y   N   I   -                    395
```

SUPPLEMENTARY FIGURE 7. Recodonized sequence of PbRpt3 cDNA optimized for translation in *Xenopus laevis*, and deduced amino acid sequence. In blue : nucleotides coding for RVxF motifs : KVTF codons AAA**GTGACTTTT** will be substituted by KATA codons AAA**GCGACTGCT**. RKIEF codons AGAAAG**ATTGAGTTC** will be substituted by RKAEA codons AGAAAG**GCAGAGGCA** for the simple mutant, and by AGAAAG**GCTGAGGCC** for the double mutant. In yellow : nucleotides coding for the four residues K188, D241, N288 and Q356 which bind or stabilize the ATP have been substituted by GCA which codes for alanine.

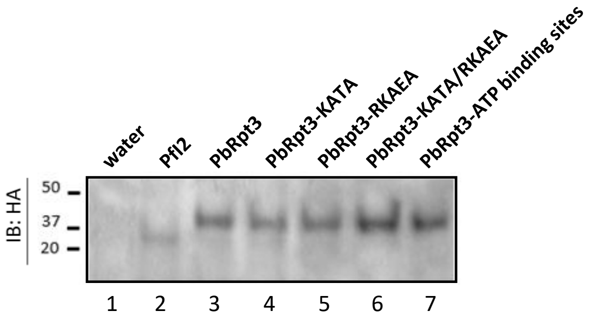

SUPPLEMENTARY FIGURE 8. PbRpt3 functional analysis using *Xenopus* oocytes model. PbRpt3 and mutated PbRpt3 proteins are expressed in *Xenopus* oocytes following the micro-injection of cRNA (60 ng in 80 nl). PfI2 is used as a positive control. Immunoblot analysis of extracts prepared from micro-injected oocytes with either water (lane 1) or cRNAs coding for PfI2 (lane 2), PbRpt3 (lane 3), PbRpt3-KATA (lane 4), PbRpt3-RKAEA (lane 5), PbRpt3-KATA/RKAEA (lane 6) or PbRpt3-ATP binding sites (lane 7) and followed by progesterone treatment (10 µM, 15 hours after micro-injection). The presence of proteins was detected using an anti-HA mAb.

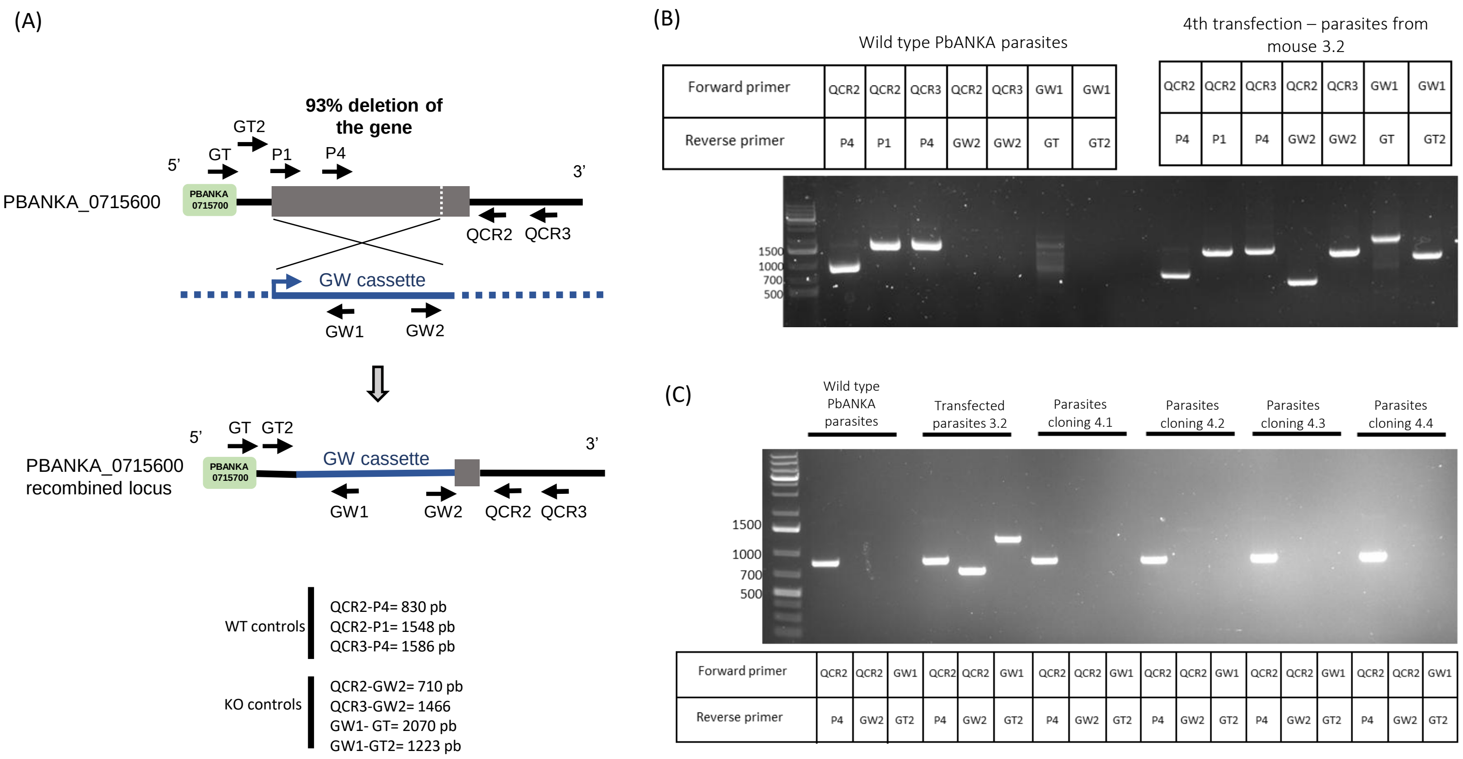

SUPPLEMENTARY FIGURE 9. PlasmoGEM construct (PbGEM-022521) and genotyping. A. The first panel shows PbRpt3 locus modification using the PbGEM-022521 construct provided by the Wellcome Sanger Institute. After linearization with NotI, the plasmid was transfected in PbANKA-GFP parasites, then injected to mice. The second panel indicates the sets of primers used and the expected fragments sizes : unmodified endogenous locus was detected using primers QCR2/P4, QCR2/P1 and/or QCR3/P4. The integration of the dhfr resistance cassette at the PbRpt3 locus was assessed using primers QCR2/GW2, QCR3/GW2, GW1/GT and/or GW1/GT2. **B.** PCR performed on wild-type (WT) parasites, or from parasites obtained in the 4th transfection (mouse 3.2). **C.** PCR performed on WT parasites, transfected parasites (mouse 3.2) or after cloning by limiting dilution in mice (mice 4.1 to 4.4).

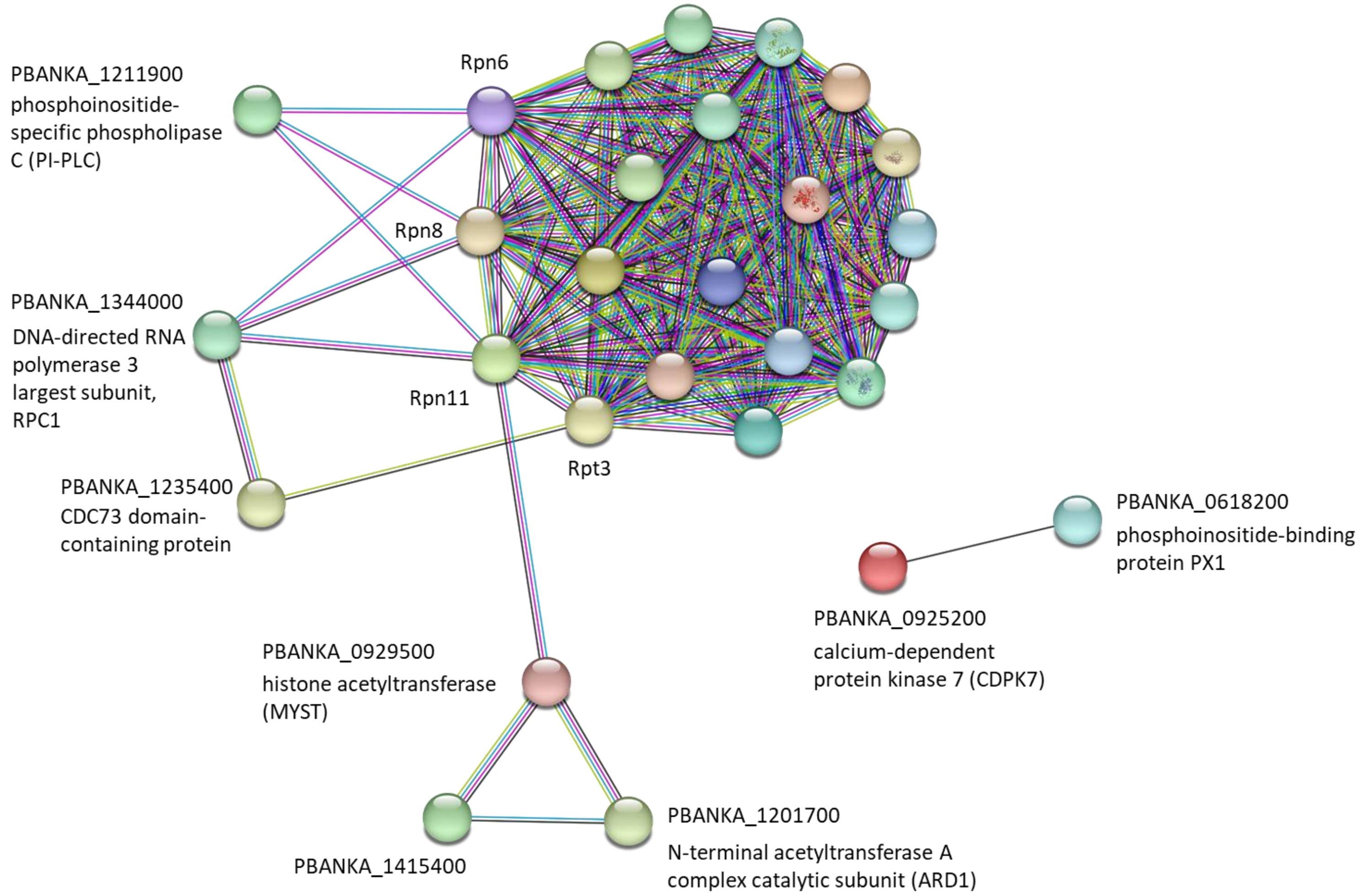

SUPPLEMENTARY FIGURE 10. STRING network visualization of PbRpt3-interacting proteins belonging to the 10 enriched GO terms (39 proteins, see Supplementary Table 3). With a protein–protein interaction enrichment *p*-value < 1.0e-16, the resulting network contained 203 functional associations among 28 proteins; the proteins with no associations to other proteins in the network were removed. For clarity, the gene Id and names of proteins belonging to the proteasome 19S were removed, except for Rpt3, Rpn6, Rpn8 and Rpn11 for which only the short names were kept. STRING analysis was performed using the 11.5 version of the software.

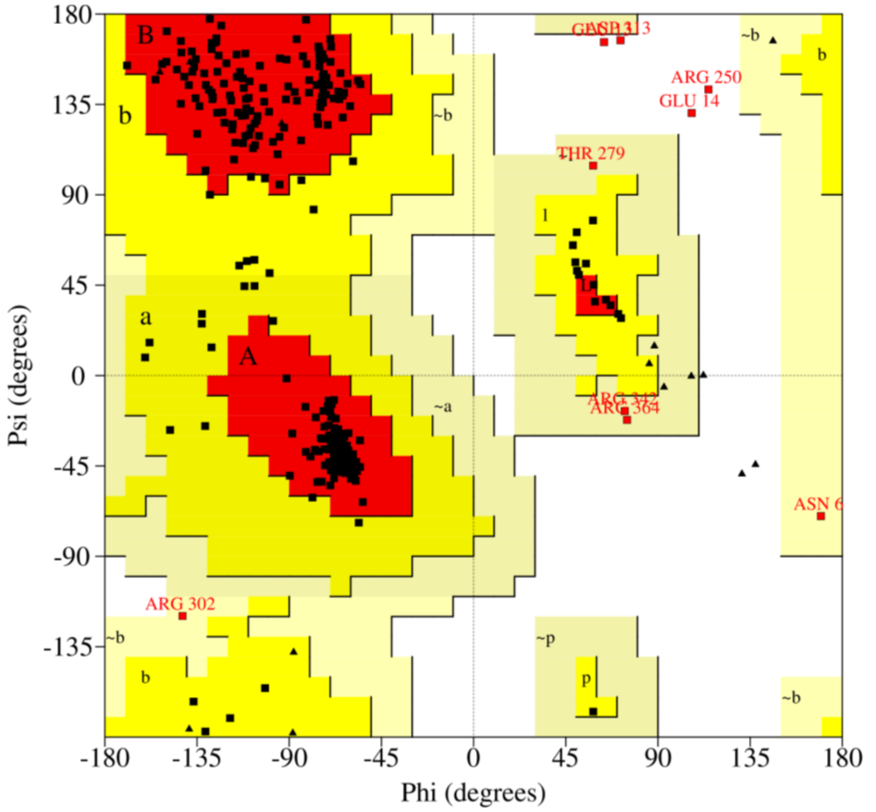

Plot statistics

Residues in most favoured regions [A,B,L]	314	87.7%
Residues in additional allowed regions [a,b,l,p]	35	9.8%
Residues in generously allowed regions [~a,~b,~l,~p]	5	1.4%
Residues in disallowed regions	4	1.1%
	----	------
Number of non-glycine and non-proline residues	358	100.0%
Number of end-residues (excl. Gly and Pro)	2	
Number of glycine residues (shown as triangles)	19	
Number of proline residues	16	

Total number of residues	395	

SUPPLEMENTARY FIGURE 11. Ramachandran plot and statistics of the PbRpt3 (PBANKA_0715600) **model**, based on the cryomicroscopic structures of PSMC4 (PDB file 6MSB – chain D).

References for Supplementary figures

1- Zander U, Hoffmann G, Cornaciu I, Marquette JP, Papp G, Landret C, Seroul G, Sinoir J, Röwer M, Felisaz F, Rodriguez-Puente S, Mariaule V, Murphy P, Mathieu M, Cipriani F, Márquez JA. Automated harvesting and processing of protein crystals through laser photoablation. Acta Crystallogr D Struct Biol. 2016 Apr;72(Pt 4):454-66. doi: 10.1107/S2059798316000954.

2-Huang X, Luan B, Wu J, Shi Y. An atomic structure of the human 26S proteasome. Nat Struct Mol Biol. 2016 Sep;23(9):778-85. doi: 10.1038/nsmb.3273.

3-Dong Y, Zhang S, Wu Z, Li X, Wang WL, Zhu Y, Stoilova-McPhie S, Lu Y, Finley D, Mao Y. Cryo-EM structures and dynamics of substrate-engaged human 26S proteasome. Nature. 2019 Jan;565(7737):49-55. doi: 10.1038/s41586-018-0736-4.

4-Hänzelmann P, Schindelin H. Structural Basis of ATP Hydrolysis and Intersubunit Signaling in the AAA+ ATPase p97. Structure. 2016 Jan 5;24(1):127-139. doi: 10.1016/j.str.2015.10.026.
